# Multivariate fetal growth trajectory modeling and its association with maternal fatty acids

**DOI:** 10.1038/s41598-025-30334-5

**Published:** 2025-12-30

**Authors:** Shuai Huang, Jia-Jia Tang, He-Bin Chi, Han-Wen Zhang, Xiao-Yuan Fan, Feng Tang, Xian-Shu Lin, Bing-Rui Yang, Hong-Bo Qi, Yin-Yin Xia, Ting-Li Han, Hua Zhang

**Affiliations:** 1 Department of Obstetrics and Gynaecology, The First Affiliated Hospital of Chongqing Medical University, Chongqing, China; 2 School of Public Health, Chongqing Medical University, Chongqing, China; 3 Department of Obstetrics and Gynecology, Women and Children’s Hospital of Chongqing Medical University, Chongqing, 401120 China; 4 Department of Obstetrics and Gynaecology, The Second Affiliated Hospital of Chongqing Medical University, Chongqing, China

**Keywords:** Fatty acids, Group, Based multi, Trajectory modeling, Quantile g, Computation, Fetal growth, Mixture analysis, Embryogenesis, Intrauterine growth, Medical research

## Abstract

Maternal circulating fatty acids are closely linked to fetal development. However, few studies have investigated the relationship between the maternal fatty acid levels at 11–14 weeks of gestation and fetal growth trajectories phenotype. A total of 655 pregnant women were selected from the Complex Lipids in Mothers and Babies cohort. Twenty fatty acids in maternal serum at 11–14 weeks of gestation were quantified by GC–MS. The trajectories of fetal head circumference (HC), biparietal diameter (BPD), abdominal circumference (AC), and femur length (FL) z-score at 11–14, 22–28, and 32–34 weeks of gestation were determined by group-based multi-trajectory modeling (GBMTM). Multinomial logistic regression and quantile-based g-computation (Qgcomp) were used to investigate the relationship between individual and mixed fatty acid exposures and the fetal growth trajectories. GBMTM identified four trajectory groups of fetal ultrasound measurements: stable falling, stable increasing, high stable increasing, and dramatically falling. The incidence of premature rupture of membranes, placental implantation, large-for-gestational-age (LGA) and macrosomia differed significantly between the four trajectory groups (*p*-values of 0.044, 0.008, < 0.001, and < 0.001, respectively). Higher maternal serum linoleic acid (LA, C18:2 n-6) and α-linolenic acid (ALA, C18:3 n-3) were associated with increased odds in the stable falling (OR = 1.004; 95% CI: 1.001, 1.008) and dramatically falling trajectory groups (OR = 1.042; 95% CI: 1.001, 1.085). Meanwhile, eicosadienoic acid (EDA, C20:2 n-6) was associated with decreased odds in the stable falling trajectory group (OR = 0.845;95% CI: 0.716, 0.998). Fetal growth trajectory classification based on GBMTM reveals associations between different trajectory groups and adverse pregnancy outcomes. In addition, serum fatty acids LA, ALA and EDA at 11–14 weeks of gestation were associated with the fetal growth trajectories phenotype.

**Supplementary Information:**

The online version contains supplementary material available at 10.1038/s41598-025-30334-5.

## Introduction

Fatty acids are a class of organic compounds widely found in the bodies of animals and plants, and they are the primary components of neutral fats, phospholipids, and glycolipids. Based on the number of double bonds in the carbon chain, fatty acids can be classified into saturated fatty acids (SFAs) and unsaturated fatty acids. Unsaturated fatty acids can further be divided into monounsaturated fatty acids (MUFAs) and polyunsaturated fatty acids (PUFAs), with the latter categorized into n-3 and n-6 fatty acids based on the position of the first double bond. These two types of fatty acids are essential for maintaining human health, as the human body cannot independently synthesize their precursors, alpha-linolenic acid (ALA, C18:3 n-3) and linoleic acid (LA, C18:2 n-6), and must obtain them from the diet. Therefore, they are also referred to as essential fatty acids. Fatty acids play an important role in living organisms. They are an important component of cell membranes and participate in cell signaling, hormone synthesis, energy storage, and energy supply^1^. It is particularly important to note that long-chain polyunsaturated fatty acids, including docosahexaenoic acid (DHA, C22:6 n-3) and eicosapentaenoic acid (EPA, C20:5 n-3), are essential for infant brain development and vision protection^2,3^. Furthermore, fatty acids are also involved in regulating inflammatory responses and immune function, and are essential nutrients for maintaining good health^4–6^. Throughout the human life cycle, the fetal period is a critical stage of metabolic programming, and the interaction between nutritional supply patterns and fatty acid metabolism during this period is particularly noteworthy^7^. This dynamic interaction is not only reflected in the maintenance of basic physiological functions, but may also have a profound impact on health through epigenetic regulation and other mechanisms^8,9^^.^

Currently, research has found that fatty acids play multiple key roles during pregnancy and infancy^10–12^. First, as a high-density energy substance, fatty acids provide important metabolic support for mothers and infants^10,13^. Secondly, in terms of structure, they are not only core components of cell membranes, but also participate in multiple physiological regulatory processes by converting into bioactive signaling molecules^12,14^. More importantly, fatty acids may also influence fetal tissue differentiation and organ morphogenesis, playing a decisive role in early embryonic development during pregnancy^15,16^. It is worth noting that this maternal–fetal nutrient transfer has a special time sensitivity, meaning that the types and amounts of fatty acids transferred through the placenta during pregnancy may produce lifelong metabolic programming effects during critical windows of fetal development^17^. Previous studies have shown that high intake of n-6 polyunsaturated fatty acids by pregnant women is negatively correlated with fetal birth weight and height^18^; whereas supplementation with n-3 PUFAs (such as DHA and EPA) has multiple positive effects, including promoting fetal abdominal circumference growth^19^, prolonging the gestational period, and reducing the risk of preeclampsia and preterm birth , among others^20,21^. It is also worth noting that the promoting effect of n-3 fatty acids on the abdominal circumference may be related to their mechanism of regulating placental lipid transport and fetal fat accumulation^22^. The above studies indicate that the balance of maternal fatty acid metabolism during pregnancy is an important factor influencing fetal growth patterns in utero. Although numerous studies have explored the effects of fatty acid supplements on birth weight, body shape, and long-term obesity risk^23^, there has been a lack of systematic research on the association between maternal circulating fatty acids in early pregnancy and multidimensional fetal growth trajectories. In addition, previous studies have mainly used single anthropometric indicators to develop fetal growth trajectories^24–26^, which lack information about the dynamic growth process of the fetus compared to models that establish combinations of multiple measurement indicators. The heterogeneity of early fetal growth requires these changes to be modeled more precisely as multiple typical growth trajectories, rather than using a single growth trajectory for each indicator.

Currently, a number of methods have been proposed to evaluate the fetal dynamic growth processes during pregnancy, among which group-based trajectory modeling (GBTM) is used to group individuals with similar developmental trajectories and classify them into a specific category^27–29^. Group-based Multivariate Trajectory Modeling (GBMTM) as an extension of the univariate GBTM, was designed to defining a trajectory group in terms of trajectories for multiple indicators not just one. This approach allows for the examination of multivariate longitudinal data, facilitating the exploration of the course and interrelationships among different clinically relevant indicators in the fetal growth process^30^. However, to the best of our knowledge, no study has investigated the relationship between changes in maternal fatty acid levels and dynamic fetal growth trajectories using the GBMTM model during pregnancy.

To address this, we used GBMTM to characterize fetal growth trajectories in the Complex Lipids In Mothers and Babies cohort (CLIMB), and assess whether the maternal fatty acids at 11–14 weeks of gestation, both individually and overall, were associated with specific growth trajectories throughout gestation.

## Methods

This cohort study was approved by the Human Research Ethics Committee of Chongqing Medical University, and written informed consent was obtained from all participants. The study followed the Strengthening the Reporting of Observational Studies in Epidemiology (STROBE) reporting guideline. Approval for the clinical trial (2,014,034) was obtained from the Ethics Committee of Chongqing Medical University.

### Study design and population

**Fig. 1:**
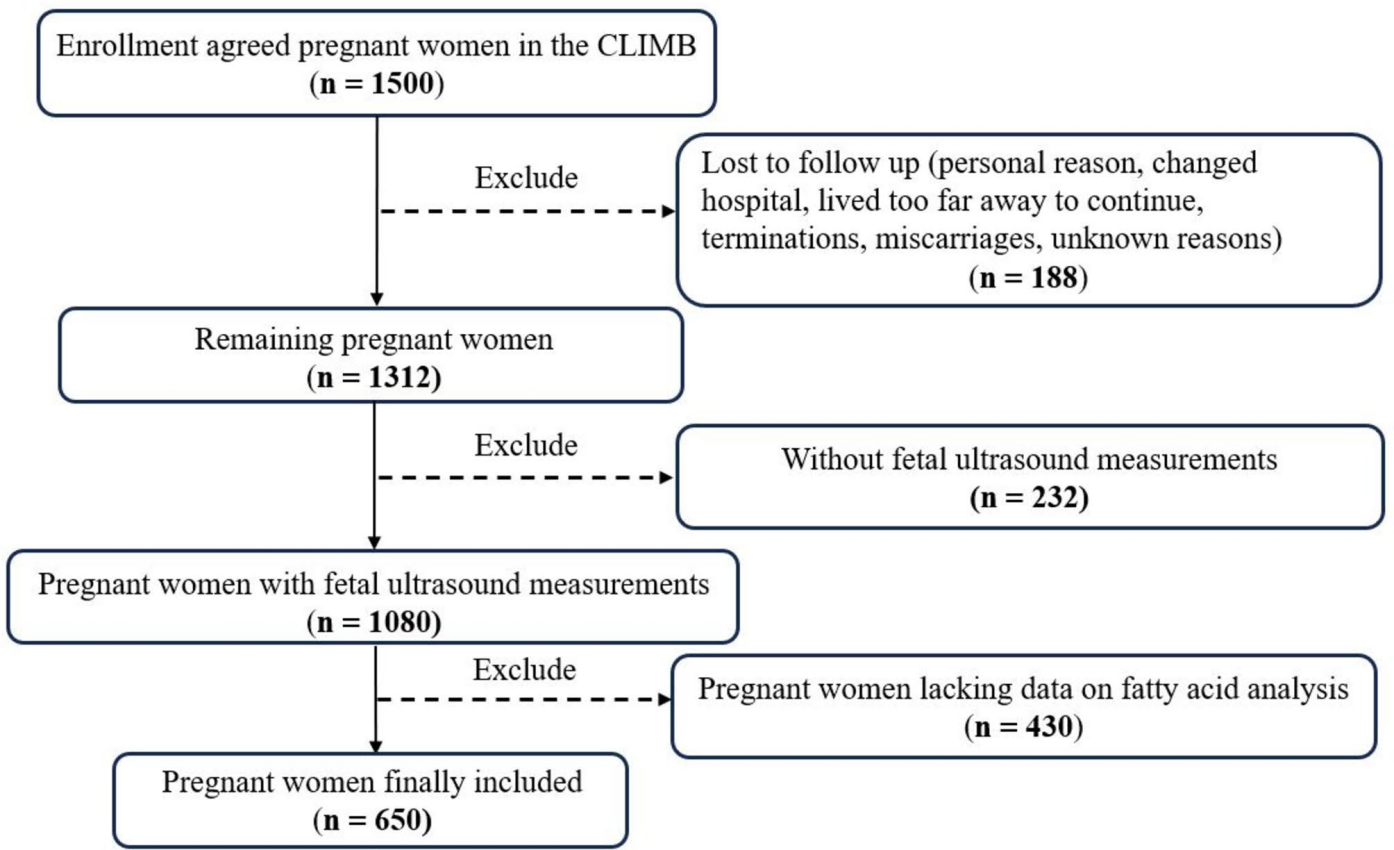
Flowchart of study participants.

This study utilizes data from the CLIMB study, conducted at the First Affiliated Hospital of Chongqing Medical University and Chongqing Health Centre for Women and Children in China from September 2015 to May 2018.The study design and protocol have been previously detailed^31^. A total of 1,500 pregnant women were recruited for the CLIMB study. After excluding pregnant women who were lost to follow-up or experienced miscarriage or stillbirth, 1,312 live births were included in the final analysis. Then, we excluded 232 pregnant women who lacked complete fetal ultrasound measurement data from three prenatal checkups. We further restricted the study population to pregnant women who had maternal serum samples from early pregnancy available for fatty acid analysis (total *n* = 655; refer to Fig. 1 for flow chart). During the recruitment process, all pregnant women were provided with detailed basic information about the study and assured the refusal to participate would not affect subsequent prenatal care. Following full disclosure, pregnant women signed an informed consent form.

### Fatty acids analysis and quantification

We measured the serum concentrations of 20 fatty acids by gas chromatography-mass spectrometry (GC–MS) (Agilent 5977A mass spectrometer/7890B gas chromatography, Agilent Technologies, USA) analysis as described previously^32^. Prior to fatty acids extraction, an internal standard, heptadecanoic acid (C17:0), was added to each sample. Fatty acid separation was conducted on a DB-23 capillary column (20 m × 0.18 mm × 0.20 μm, Agilent Technologies, USA). Chromatographic peak heights for each fatty acid were determined using Agilent ChemStation software (version 2.6). Levels of individual serum fatty acids were first normalized to the internal standard and then quantified to absolute concentrations using calibration curves derived from the corresponding chemical standard.

### Anthropometric measurements

Data on fetal growth and development was obtained from medical records. According to the maternal health handbook, pregnant women are required to undergo multiple ultrasound examinations during pregnancy, which are performed by specialized ultrasonographers who issue paper reports. Fetal ultrasound measurements such as head circumference (HC), biparietal diameter (BPD), abdominal circumference (AC) and femur length (FL) are taken at 11–14, 22–28, and 32–34 weeks of gestation. These measurements followed the Chinese Ultrasonographers’ Association (CUDA) Guidelines for Fetal Ultrasound Scanning Operations, 2012 edition. To analyze fetal growth, the Z-scores of fetal ultrasound measurement indicators in relation to gestational age and according to sex were calculated using the international standard developed by the INTERGROWTH-21st standards^33,34^. We used the free tool available on the Intergrowth-21st website^35^. The gestational age was calculated based on the last menstrual period (LMP) and confirmed by an ultrasound scan before the 16th week of gestation. If there was a difference of seven or more days between the date of the last menstrual cycle and the date of the ultrasound, or if the last menstrual cycle was uncertain, the earliest ultrasound measurement data should be used to determine the date^36^. All adverse pregnancy outcomes for mothers are derived from medical records and the definitions of some adverse pregnancy outcomes are as follows: Gestational diabetes mellitus (GDM) is typically defined as hyperglycemia that is diagnosed or develops during pregnancy^37^; Prematurity is defined as a delivery or birth at a gestational age less than 37 weeks^38^; Preeclampsia refers to high blood pressure and proteinuria occurring after 20 weeks of pregnancy, and may be accompanied by symptoms such as headache, blurred vision, nausea, vomiting, and upper abdominal discomfort^39^; Fetal distress refers to the appearance of signs of oxygen deprivation in the fetus within the uterus, which poses a threat to the health and life of the fetus^40^; Cesarean delivery refers to a surgical delivery method used when a woman is unable to give birth vaginally^41^; Premature rupture is the rupture of gestational membranes after 37 weeks but before the process of labour begins^42^; Chorioamnionitis is an infection that can occur before labor, during labor, or after delivery^43^; Anemia of pregnancy refers to a decrease in the concentration of red blood cells or hemoglobin in the blood^44^; Hypothyroidism during pregnancy is defined as overt hypothyroidism (elevated serum thyroid-stimulating hormone [TSH] and low serum free thyroxine [FT4])^45^; Abnormal fetal position refers to any fetal position other than the optimal position for vaginal delivery (occiput anterior position) ; Nuchal cord refers to the umbilical cord wrapping around the fetus’s neck during childbirth; Placenta implantation refers to the placental villi penetrating part of the uterine wall muscle layer during early pregnancy^46^; Oligohydramnios is a condition characterized by reduced amniotic fluid volume for gestational age^47^; Low birth weight (LBW) is defined as a birth weight of less than 2500 g^48^; Small for gestational age (SGA) was defined as birth weight below the 10th percentile for gestational age^49^; Large for gestational age (LGA) was defined as birth weight above the 90th percentile for gestational age; Macrosomia generally refers to a fetus with a birth weight greater than 4000 to 4500 g (9 to 10 pounds)^50^.

### Covariates

According to previous literature and directed acyclic graph (DAG) (eFigure 1 in Supplement), all models were adjusted for the following potential confounders: maternal Socio-economic status, smoking /drinking, body mass index (BMI), maternal age and fetal sex as covariates in our analysis^51^. The DAG was constructed a priori using published causal models of maternal diet and fetal growth^52^.Self-reported data on maternal characteristics and behaviors were obtained from the mothers during pregnancy or follow-up visits, including maternal age at delivery (continuous years), maternal education, maternal occupations(full-time work, part-time work and student), maternal pre-pregnancy BMI (continuous, kg/m^2^), active or passive smoking during pregnancy (yes/no), maternal income (< ¥ 2000/month, < ¥ 4000/month, < ¥ 7000/month, < ¥ 10,000/month), maternal marriage (yes/no) and maternal ethnic groups. Other important covariates were obtained from hospital medical records, including child sex (male, female) and maternal weight at delivery (continuous, kg).

### Statistical analysis

The GBMTM was utilized to describe the growth trajectories of AC, HC, BPD, and FL. GBMTM, a form of finite mixture modeling, employs trajectory groups as a statistical tool for approximating unknown trajectories of population members. This modeling is applied to identify individuals with similar developmental trajectories within the population and to classify them into specific groups. To obtain the developmental trajectories of the study subjects, it is necessary to establish the number of trajectory groups and patterns. Usually, 2–6 trajectory model groups were constructed, each fitted linearly, squarely, and cubically. The optimal model is selected by evaluating the fit metrics of different models and the professional interpretability of trajectory group morphology. Various indicators were used to evaluate the adequacy of model fit in GBMTM, including the Bayesian Information Criterion (BIC), average a posteriori probability (Avepp), and the proportion of each trajectory group. Model selection plays a vital role in GBMTM analysis, with the BIC commonly employed to determine the optimal model fit. Specifically, the optimal number of trajectory groups and shapes is based on BIC values, with higher values indicating a better simulation fit. The closer the Avepp to 1, the higher the level of conformity within the trajectory is considered to be, with a threshold > 0.7 indicating a strong degree of conformity. The proportion of each trajectory group is generally no less than 5%^30,53,54^^.^

Based on the above guidelines, the growth trajectory pattern of the hybrid model was established based on four indicators of fetal BPD, HC, AC, and FL. Based on the above criteria, the optimal number of trajectory groups for the hybrid model was determined to be four, with a BIC value of -17,110.42. The sample share of each of the four groups was 19.32, 56.28, 15.39 and 8.99%, with an Avepp of 0.86, 0.90, 0.87, 0.95. Finally, we described the differences in demographic characteristics among the mixed model trajectory.

P-P plots and Shapiro–Wilk W tests were used to assess the distribution of the data. For variables that followed a normal distribution, the mean ± standard deviation (x̄ ± SD) was reported; for variables that did not follow a normal distribution, the median and interquartile range (IQR) were used for description. Fetal ultrasound measurement parameters are continuous variables that follow a normal distribution, so their characteristics are described using the mean and standard deviation. For adverse pregnancy outcomes in mothers of each trajectory group, which are dichotomous variables, the frequency (n) and percentage are used for description, and the chi-square test or Fisher’s exact test is used to analyze the incidence of adverse pregnancy outcomes between trajectory groups. Additionally, given that serum fatty acids exhibit a skewed distribution, natural logarithmic transformation was applied prior to statistical analysis. The Mann–Whitney U test was used for intergroup comparisons of continuous variables, while the chi-square test or Fisher’s exact test was employed to compare demographic characteristics between different groups for categorical variables.Multinomial logistic regression was used to investigate the association between the levels of the 20 detected serum fatty acids and fetal growth trajectories. We adjusted for potential confounding factors, with the minimum necessary covariates determined by the DAG including maternal age, pre-pregnancy body mass index, household income, maternal years of education, occupation, smoking and alcohol consumption, marital status, and Han ethnic groups. Odds ratios (OR) and their 95% confidence intervals (CI) were estimated in the analysis. To address potential selection bias from inclusion criteria, inverse probability of treatment weights (IPTW) were generated for each anthropometric measure based on an assessment of baseline data (including maternal education, smoking, BMI, etc.) for all women in the CLIMB study. All regression models assessing the relationship between fetal fatty acid levels and growth trajectory groups were weighted using IPTW to enhance the robustness of the results^55^. Additionally, to assess the potential influence of fetal sex on the results, this study conducted a sex-stratified analysis, evaluating heterogeneity through the p-value of the interaction term between fatty acids and fetal sex in the regression model.

In addition, to assess the joint effect of a mixture of twenty fatty acids on fetal growth trajectories, we used a quantile-based g-computation (Qgcomp). The use of Qgcomp allows for easy interpretation of the overall mixture effect because this approach combines the inferential simplicity of weighted quantile sums with the flexibility of g-computations, a causal effect estimation method^56^. In the analysis, the parameter q (representing the specified quantile) was set to 4. To determine the CIs, 100 bootstrap iterations were completed.

In the sensitivity analysis, we addressed the sex-specific effects of maternal fatty acids on fetal growth trajectories by stratifying by sex. In addition, we used the IPTW technique to balance for confounders as well as General Additive Modeling (GAM) to test nonlinearity using penalized smoothing regression splines with a degree of 3. A *p*-value less than 0.05 for spline was considered a departure from linearity.

The GBMTM model was constructed using the PROC TRAJ module in SAS software. The multinomial logistic regression model, Qgcomp model, and GAM model were constructed using the “VGAM,” “dplyr,” “qgcomp,” and “mgcv” packages in R Studio (version 4.4.0), respectively. Other statistical analyses were performed using SAS (version 9.4, SAS Institute Inc.) and SPSS software (version 22.0, IBM). Figures were created using the “ggplot2” R package in R software (version 4.4.0, RStudio). All tests were two-sided, with a significance level of 0.05.

## Results

This study summarized and compared the basic information of 650 pregnant women. However, no statistically significant differences were found between the four fetal growth trajectory group, specific results are shown in Table 1. In addition, ultrasound measurement parameters related to the fetus (including AC, HC, BPD, and FL) are shown in Table 2.

**Table 1:** Basic demographics of the study participants.

Selected characteristics	stable falling (*n* = 116)	stable increasing (*n* = 394)	high stable increasing (*n* = 92)	dramatically falling (*n* = 53)	*p*-value
Age, years	28.85 ± 3.44	28.6 4 ± 3.61	28.96 ± 3.85	28.51 ± 3.88	0.820
BMI (kg/m^2^)	21.02 ± 2.53	21.52 ± 3.05	21.98 ± 3.01	21.06 ± 2.75	0.086
Income (%)					0.918
< 2000 ¥ / month	20 (17.2%)	78 (19.8%)	16 (17.4%)	8 (15.1%)	
< 4000 ¥ / month	40 (34.5%)	137 (34.8%)	33 (35.9%)	23 (43.4%)	
< 7000 ¥ / month	38 (32.8%)	130 (33.0%)	28 (30.4%)	17 (32.1%)	
< 10,000 ¥ / month	18 (15.5%)	49 (12.4%)	15 (16.3%)	5 (9.4%)	
Education, years	15.92 ± 1.97	15.52 ± 1.99	15.48 ± 2.06	15.66 ± 1.48	0.238
Occupations (%)					0.420
full time work	27 (23.3%)	89 (22.6%)	17 (18.5%)	11 (20.8%)	
part time work	60 (51.7%)	188(47.7%)	49 (53.3%)	33 (62.3%)	
student	29 (25.0%)	117 (29.7%)	26 (28.3%)	9 (17.0%)	
No smoking /drinking (%)	116 (100.0%)	393 (99.7%)	90 (97.8%)	53 (100.0%)	0.069
Marriage (%)	115(99.1%)	390 (99.0%)	89 (96.7%)	53 (100.0%)	0.252
Han ethnic groups (%)	113 (97.4%)	385 (97.7%)	89 (96.7%)	52 (98.1%)	0.945
Docosahexaenoic acid	69.96 ± 20.62	71.37 ± 24.48	70.42 ± 21.18	69.00 ± 23.96	0.868
Eicosapentaenoic acid	10.58 ± 8.11	10.84 ± 8.37	10.36 ± 7.17	8.57 ± 5.31	0.277
α-linolenic acid	27.83 ± 11.60	29.14 ± 14.42	29.40 ± 14.02	28.19 ± 11.19	0.777
Docosapentenoic acid	11.18 ± 4.30	11.40 ± 4.19	11.31 ± 3.59	10.07 ± 2.91	0.164
γ-linolenic acid	8.78 ± 5.60	9.06 ± 5.85	7.86 ± 4.30	7.02 ± 2.99	0.059
Arachidonic acid	174.43 ± 53.12	177.72 ± 50.24	172.12 ± 43.41	168.33 ± 42.44	0.493
Docosatetraenoic acid	6.47 ± 2.11	6.60 ± 2.19	6.45 ± 1.63	6.04 ± 1.50	0.314
Eicosadienoic acid	9.46 ± 3.36	9.94 ± 3.52	10.08 ± 3.59	9.98 ± 3.34	0.541
Eicosatrienoic acid	73.63 ± 30.48	78.37 ± 34.87	74.56 ± 26.69	71.36 ± 26.31	0.277
Linoleic acid	745.89 ± 178.64	742.36 ± 159.78	763.84 ± 167.07	743.97 ± 142.79	0.728
Arachidic acid	5.88 ± 1.71	6.16 ± 1.78	6.04 ± 1.58	6.12 ± 1.60	0.461
Docosanoic acid	13.84 ± 4.55	14.62 ± 4.89	14.76 ± 4.67	14.95 ± 3.85	0.352
Eicosaenoic acid	5.41 ± 4.03	5.55 ± 2.49	5.56 ± 2.78	4.85 ± 1.66	0.387
Hexadecenoic acid	25.85 ± 12.80	25.26 ± 12.40	23.87 ± 9.35	22.06 ± 8.28	0.183
Hexadecanoic acid	472.34 ± 106.66	470.94 ± 110.16	465.78 ± 100.85	448.37 ± 86.78	0.512
Lignoceric acid	10.31 ± 3.94	10.67 ± 3.90	10.97 ± 3.81	11.25 ± 3.24	0.435
Octadecenoic acid	379.36 ± 96.31	378.60 ± 96.57	372.96 ± 85.52	351.14 ± 82.30	0.237
Octadecanoic acid	147.88 ± 33.86	150.60 ± 31.80	152.83 ± 31.86	147.99 ± 24.70	0.665
Tetracosenic acid	34.98 ± 13.51	36.35 ± 13.26	35.88 ± 12.83	33.54 ± 10.59	0.433
Tetradecanoic acid	13.26 ± 8.17	13.24 ± 7.90	12.29 ± 5.50	12.64 ± 6.96	0.707

Continuous variables are represented by mean ± standard deviation (x̄ ± SD), categorical variables are represented by number (percentage); *p*-values are for statistical comparison between the multiple groups (Chi-square test, or Fisher’s exact test).

**Table 2:** Ultrasonographic parameters of fetuses at 11–14,22–28 and 32–34 weeks of gestation in the CLIMB study.

Ultrasound measurement values (mm)	11–14 weeks		22–28 weeks		32–34 weeks	
	(*n* = 1080)	(*n* = 655)	(*n* = 1080)	(*n* = 655)	(*n* = 1080)	(*n* = 655)
Biparietal diameter	19.19 ± 3.68	18.99 ± 3.53	59.11 ± 2.62	59.27 ± 2.46	81.00 ± 3.11	81.06 ± 3.09
Head circumference	72.37 ± 14.06	71.23 ± 14.10	218.72 ± 7.99	219.24 ± 7.34	292.46 ± 15.71	293.17 ± 9.45
Abdominal circumference	61.13 ± 11.11	60.52 ± 10.49	192.99 ± 8.78	193.07 ± 8.41	281.27 ± 17.44	281.87 ± 12.51
Femur length	7.86 ± 2.83	7.68 ± 3.66	42.29 ± 1.89	42.50 ± 1.85	61.40 ± 2.63	61.45 ± 2.40

Values are expressed as mean ± standard deviation (x̄ ± SD); *n* = 1080 represents the total number of participants in the cohort with fetal ultrasound measurements, and *n* = 655 represents the final number of participants with fetal ultrasound measurements included in this study.

**Fig. 2:**
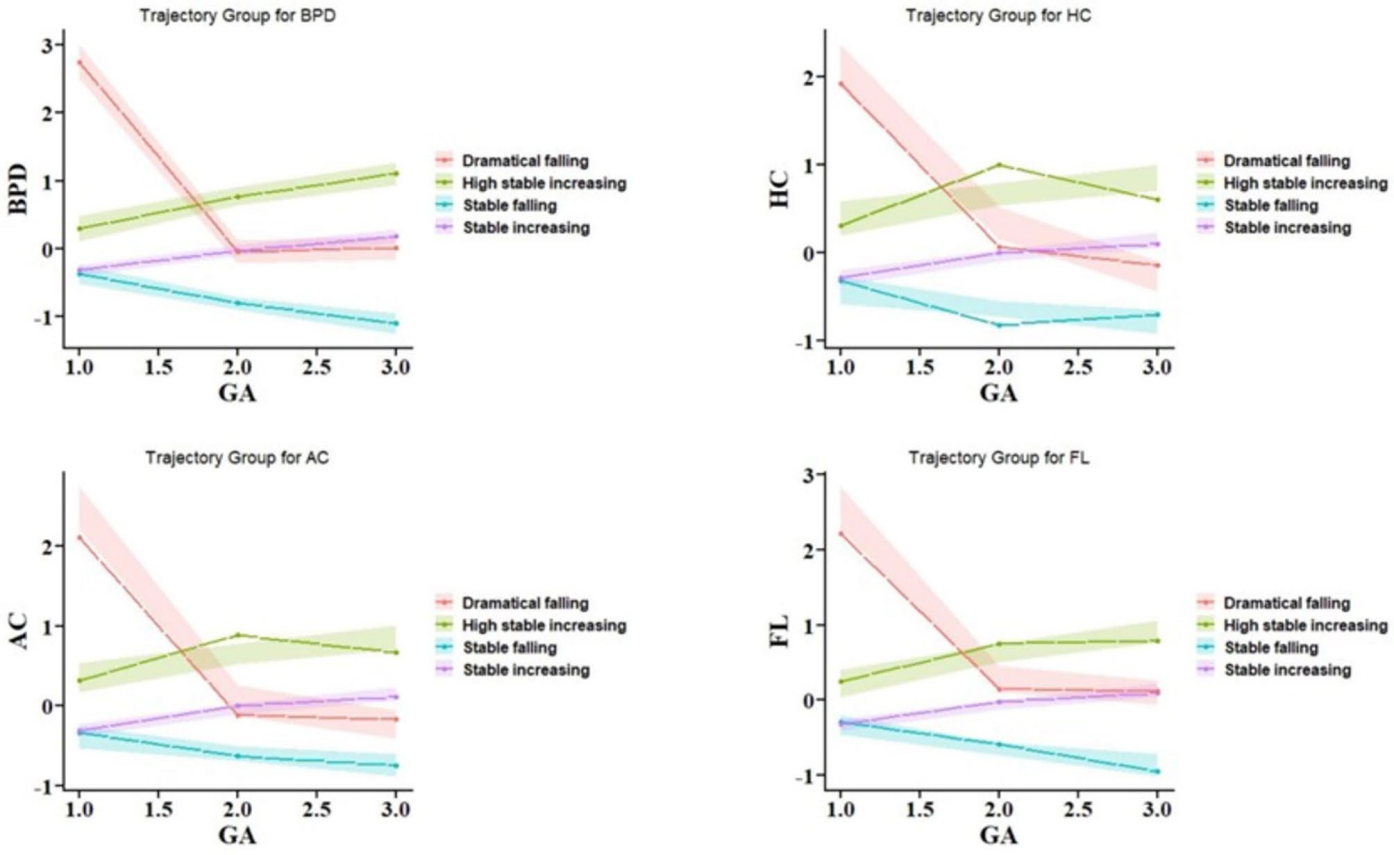
Trajectories based on four fetal indicators: BPD, HC, AC and FL. (**A**) The individual-indicator plot produced by the Group-Based Multi-Trajectory Model (GBMTM). (**B**) Stratified trajectories plot for male and female fetuses.

In this study, the GBMTM identified four trajectory groups for the above four metrics (Fig. 2). The four trajectory groups were categorized as stable falling, stable increasing, high stable increasing, and dramatically falling. Of particular interest is the stable increasing group, which exhibited a consistent trend and represented a substantial portion of the study population. Consequently, this group was selected as the reference group. The BIC used for model selection and posterior probability for model goodness of fit can be found in supplementary material (eTable1-2 in Supplement).

The incidence of adverse pregnancy outcomes was compared among four distinct fetal growth trajectory groups (eTable 3 in Supplement). Statistically significant differences were observed in the rates of preterm rupture of membranes (*p* = 0.044), placental implantation (*p* = 0.008), LGA (*p* < 0.001), and macrosomia (*p* < 0.001) across the groups. In contrast, no significant differences were identified among the groups for other adverse outcomes, including GDM, hypertensive disorders of pregnancy, and preterm labor.

First-trimester maternal serum concentrations of 20 fatty acids are shown in Table 3. Among these, DHA, EPA, ALA, and docosapentenoic acid belong to the n-3 PUFAs, while γ-linolenic acid, arachidonic acid, docosatetraenoic acid, eicosadienoic acid, eicosatrienoic acid, and LA are n-6 PUFAs. Additionally, lignoceric acid and arachidic acid are saturated fatty acids (Table 3).

**Table 3:** Fatty acid concentrations (mg/L) in maternal serum during early pregnancy in the CLIMB study.

Fatty acids	Concentration(mg/L)						
	Min	Max	5th	25th	50th	75th	95th
Docosahexaenoic acid (C22:6 n-3)	24.99	204.74	39.11	55.99	67.64	81.72	111.47
Eicosapentaenoic acid (C20:5 n-3)	1.26	86.55	3.37	5.38	8.25	13.34	26.68
α-linolenic acid (C18:3 n-3)	7.68	121.52	13.13	19.33	26.08	34.17	55.20
Docosapentenoic acid (C22:5 n-3)	3.40	32.63	6.27	8.60	10.34	13.28	19.71
γ-linolenic acid (C18:3 n-6)	1.32	43.69	2.79	5.08	7.38	10.72	18.45
Arachidonic acid (C20:4 n-6)	51.57	387.91	102.58	140.30	171.21	203.95	262.89
Docosatetraenoic acid (C22:4 n-6)	2.67	17.74	3.63	5.17	6.15	7.55	10.42
Eicosadienoic acid (C20:2 n-6)	3.66	30.33	5.37	7.36	9.29	11.62	16.75
Eicosatrienoic acid (C20:3 n-6)	20.44	283.22	37.33	53.58	70.09	92.17	133.35
Linoleic acid (C18:2 n-6)	374.08	1587.78	505.48	635.31	726.27	833.93	1035.96
Arachidic acid (C20:0)	2.53	14.09	3.73	4.95	5.89	6.89	9.26
Docosanoic acid (C22:0)	4.70	40.24	8.82	11.54	13.77	16.58	23.53
Eicosaenoic acid (C20:1 n-9)	1.84	33.94	2.80	3.87	4.77	6.34	10.27
Hexadecenoic acid (C16:1 n-7)	5.74	101.05	11.32	17.22	22.54	29.62	49.28
Hexadecanoic acid (C16:0)	254.12	972.90	318.61	389.04	457.76	531.82	665.38
Lignoceric acid (C24:0)	3.24	31.22	5.75	8.11	10.05	12.53	17.57
Octadecanoic acid (C18:0)	73.30	305.26	101.85	130.56	148.51	167.81	204.70
Octadecenoic acid (C18:1 n-9)	179.12	798.56	251.60	308.69	361.70	431.20	549.08
Tetracosenic acid (C24:1 n-9)	11.57	107.91	18.70	26.64	33.43	42.61	60.38
Tetradecanoic acid (C14:0)	2.04	50.98	4.95	7.86	10.88	15.91	28.62

Min, minimum; Max, maximum concentration; 5th/25th/50th/75th/95th, percentiles of distribution, the 50th percentile represents the median concentration. Data reflect measurements from maternal serum samples collected during early pregnancy (gestational weeks 10–14).

**Fig. 3:**
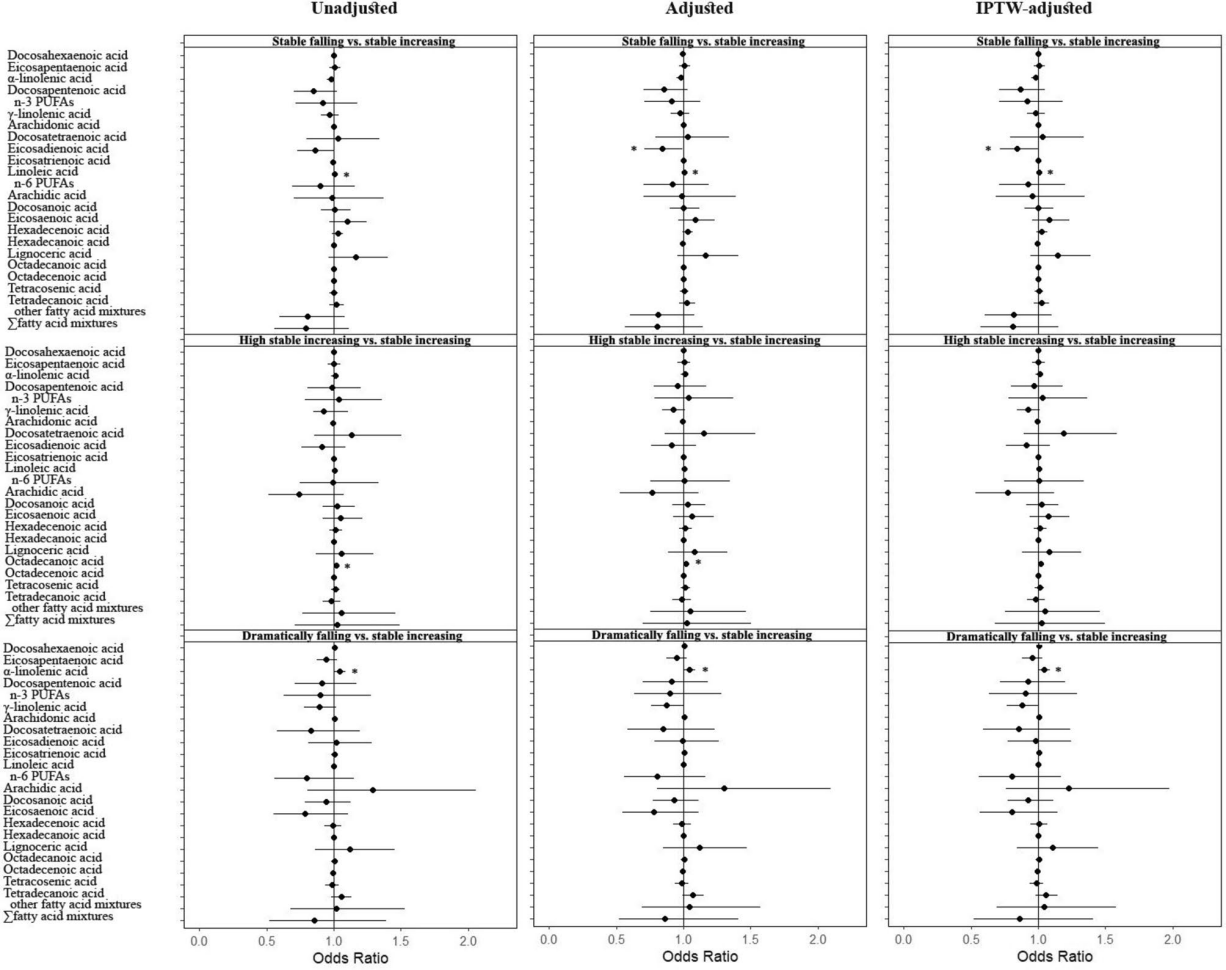
OR and 95% CI for fatty acids in each trajectory group based on each doubling of serum fatty acid levels (mg/L) in early pregnancy in logistic regression models for the CLIMB study.

The associations between serum fatty acids and fetal growth trajectories in early pregnancy are shown in figure (Fig. 3, numeric values are presented in eTable 4 in Supplement). Firstly, we observed that the level of LA was positively associated with the stable falling trajectory group (OR = 1.004; 95% CI: 1.001, 1.007), and after adjusting for covariates and using IPTW analysis, LA remained positively associated with the stable falling trajectory group (OR = 1.004; 95% CI. 95% CI: 1.001, 1.008); the original no association for EDA (OR = 0.858; 95% CI: 0.729, 1009), after adjusting for covariates and using IPTW analysis, EDA (OR = 0.845; 95% CI: 0.716, 0.998) was negatively correlated with the stable falling trajectory group, with Qgcomp weights of 0.045 and − 0.027 for LA and EDA, respectively (eTable 5 in Supplement). In addition, Octadecanoic acid was positively correlated with the highly stable increasing trajectory group, but this association disappeared after using IPTW analysis. Finally, ALA was positively correlated with the dramatically falling trajectory group (OR = 1.045; 95% CI: 1.003, 1.088), which remained positively correlated after adjusting for covariates and using IPTW analysis (OR = 1.042; 95% CI: 1.001, 1.085). Qgcomp model showed no statistical significance between n-3 PUFAs, n-6 PUFAs, other fatty acids, and serum twenty fatty acid mixtures with any of the three trajectory groups.

**Fig. 4:**
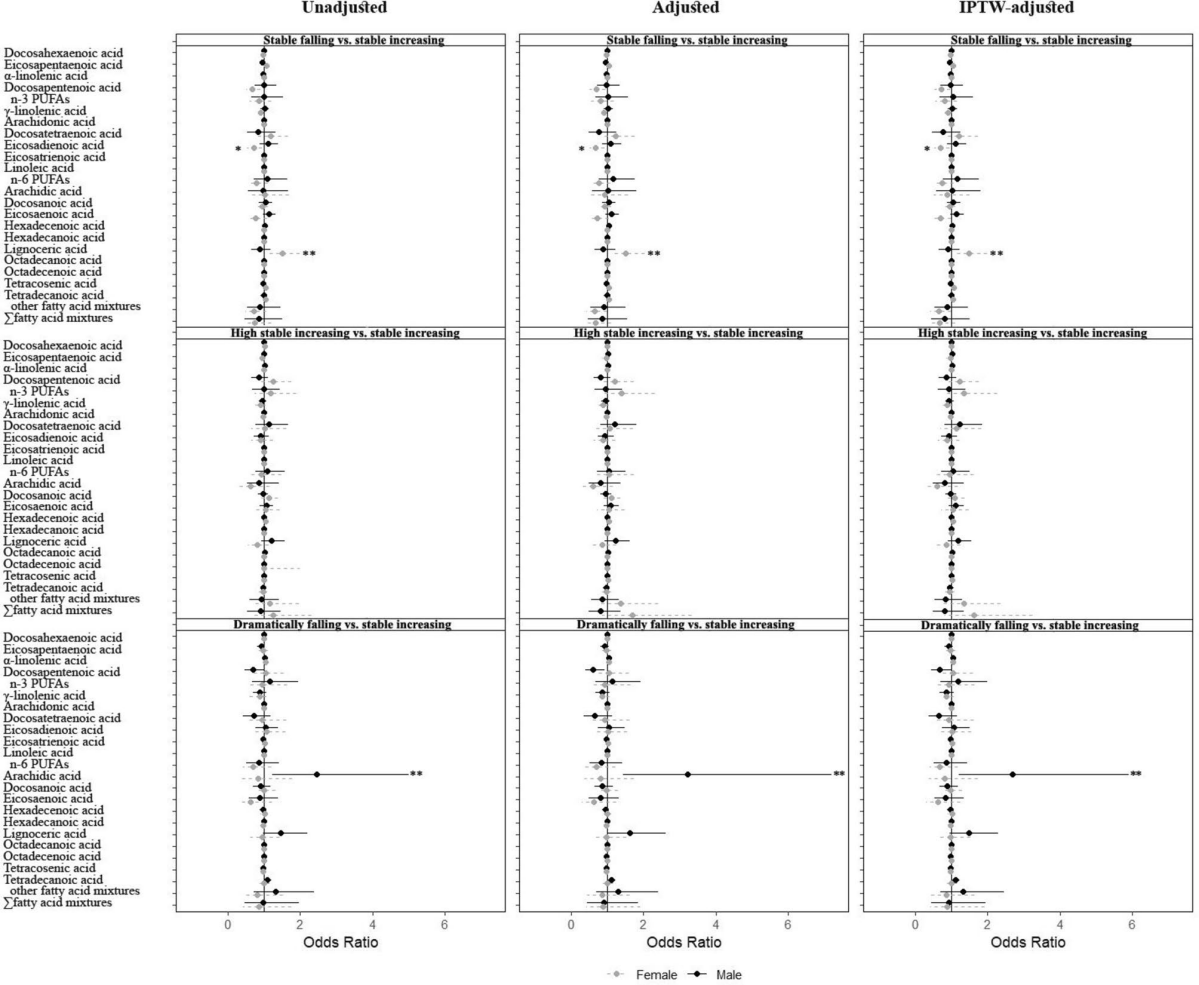
Stratified analysis by sex using logistic regression models in CLIMB Cohort. The solid line represents males, and the dashed line represents females. Lines with “*” mean a *p* < 0.05 for the comparison between males and female.

In sex-stratified analyses (Fig. 4, numeric values are presented in eTable 6 in Supplement), the most significant differences between males and females were mainly in EDA, lignoceric acid, and arachidic acid. For example, EDA (OR = 0.714; 95% CI:0.532,0.958) was negatively correlated with the female fetal stable falling trajectory group, and lignoceric acid (OR = 1.483; 95% CI:1.105,1.990) was positively correlated with the female fetal stable falling trajectory group; and arachidic acid (OR = 2.684; 95% CI:1.221,5.990) was positively associated with the male fetal dramatically falling trajectory group, with *p*-values of 0.026, 0.029, and 0.043 for the interactions, respectively(eTable 6 in Supplement). Initially, no statistically significant difference was observed in the association of tetracosenic acid with the female fetal stable falling trajectory group. However, upon adjusting for covariates and employing IPTW in the analysis, tetracosenic acid (OR = 1.061; 95% CI: 1.005, 1.119) exhibited a positive association with this group, and the *p*-value for the interaction was 0.040 (eTable 6 in Supplement). Qgcomp model showed that the other ten fatty acid mixtures, except n-3 and n-6 PUFAs, were negatively associated with the female fetal stable falling trajectory group (eTable 6 in Supplement).

In GAM (eTable 8 in Supplement), when adjusting for maternal age, maternal education, BMI, Income, Occupations, Smoking, Marriage, and Ethics groups, we did not find that ALA, LA and EDA had a nonlinear relationship with the stable falling trajectory group. After performing sex-stratified analyses, we also did not find that EDA, lignoceric acid, and tetracosenic acid were nonlinearly related to the female fetus stable falling trajectory group, but that ALA was nonlinearly related to the dramatically falling trajectory group, as well as Arachidic acid was nonlinearly related to the male fetus dramatically falling trajectory group, with corresponding *p*-values of 0.046 and 0.004, respectively. Despite the nonlinear relationship between ALA and the dramatically falling trajectory group and Arachidic acid and the dramatically falling trajectory group, their spline curves showed a monotonic trend (eFigure 2–4 in Supplement).

## Discussion

This study integrated the dynamic changes in four ultrasound parameters—fetal HC, BPD, AC, and FL applied GBMTM for the first time to reveal the group heterogeneity of fetal growth during pregnancy. We investigated the association between fatty acids in maternal serum and different fetal growth trajectories in early pregnancy. Associations between partial fatty acids and groups of fetal stable falling and dramatically falling growth trajectories were observed. In addition, we performed sex-stratified analyses, which showed that the associations between some fatty acids and fetal growth trajectories may be influenced by fetal sex.

Previous studies on fetal growth trajectories have utilized traditional methods to model different fetal growth indicators. For instance, trajectories of four ultrasound parameters (BPD, AC, HC, FL) were modeled using a cubic spline linear mixed model26; a general additive model was used for HC and HV data^57^; and a finite mixed model was constructed using repeated HC z-score measurements^58^. With the introduction of GBTM in 2001, it has become widely used in studying fetal growth trajectories^59^. The Raine study, for example, used GBTM with ultrasound fetal biometrics (AC, FL, HC) to establish growth trajectories, identifying groups of seven AC, five FL, and five HC growth trajectories 60. Since these studies have modeled several fetal indicators separately, ignoring the overall fetal growth, some other researchers have attempted to model fetal growth trajectories by converting multiple indicators of fetal growth into estimates of fetal weight (EFW) ^60,61^. Nagin proposed that GBMTM, by modeling multiple indicators jointly, provides a more comprehensive analytical framework^30,59^. For example, in the LIFECODES Fetal Growth Study, GBMTM was applied to HC, AC, and FL z-scores to identify four multivariate growth trajectories in fetuses: catch-up growth, growth of AC proportional to FL, growth of AC disproportionate to FL, and sustained growth^62^. This study provides a good insight into the link between the heterogeneity of fetal growth trajectories and concurrently developing biometric parameters. Our study integrated four fetal biometric parameters (BPD, AC, HC, FL) into the GBMTM model for a comprehensive description of fetal growth dynamics. The results showed that most fetuses exhibited a stable increase in growth trajectory, while a few showed a stable fall or an increase followed by a dramatic fall. It has been suggested that different fetal growth trajectories may in turn be associated with different growth patterns after birth as well as diseases^63,64^. Previous studies and our findings suggest that high stable increasing in fetal growth trajectory may be associated with an increased risk for the development of a large-for-gestational-age infant and macrosomia^65,66^, while placental implantation anomalies and preterm rupture of membranes may be associated with a dramatically falling trajectory group^67^. Placental implantation abnormalities, including shallow placental implantation, deep placental implantation, or placenta previa, are often associated with placental insufficiency, which impairs nutrient and oxygen exchange between the mother and fetus, leading to a dramatically falling in fetal growth^68,69^. The results of this study further support this view, suggesting that in clinical practice, it is important to strengthen monitoring of fetal growth in pregnant women, particularly focusing on the dynamic changes in fetal ultrasound measurement indicators, to enable early identification of adverse fetal growth trajectories. In short, these findings have certain clinical significance for guiding fetal growth and development during pregnancy, especially in the prevention of greater-than-gestational-age babies, macrosomia, premature rupture of membranes, and placental implantation. Therefore, the GBMTM model in our study provides a way to phenotype individual growth trajectories and reveals population heterogeneity in fetal growth, offering insights into the multifactorial interactions influencing fetal growth^70^.

In this study, we observed an inverse association between maternal serum LA and EDA (n-6 PUFAs) and the stable falling trajectory group. These findings support the hypothesis that there is a linear relationship between fatty acids and intrauterine fetal growth and development^71^^.^ Including maternal high LA diets may alter the placental fatty acid composition, affect the expression of inflammatory proteins and nutrient transporter proteins, and have harmful outcomes for the offspring^72^; increased maternal dietary intake of LA may have significant impacts on fetal development and long term consequences for the offspring, including an increased likelihood of future metabolic and psychiatric disorders^73^; and, fetal growth restriction may also be associated with maternal LA metabolism^74^. In addition, several animal studies have also shown that dietary high LA intake may adversely affect birth outcomes in mothers and offspring^75–77^^.^ Moreover, LA can be elongated to EDA in the human body and increased maternal LA levels provide more substrates for the endogenous synthesis of EDA^78^. Since the different effects of EDA on proinflammatory mediators may be the result of a negative feedback mechanism that prevents prolonged inflammation and EDA is less proinflammatory than LA, it is possible that this unusual n-6 PUFA may reduce LA-induced proinflammatory effects and thus reduce the risk of inflammation^79^. However, given the limited amount of literature available on the effects of EDA on fetal growth, more research is needed on this issue.

Apart from LA and EDA, we found a association between ALA (n-3 PUFAs) and the dramatically falling trajectory group of fetal growth. This group’s growth pattern suggests that increased maternal ALA levels in early pregnancy may lead to a high rate of fetal growth and development in early gestation but a gradual slowing down of the growth rate in mid-and late gestation. As shown in a study from the American Fetal Growth Study-Single Case Cohort, ALA and DHA were positively correlated with all fetal growth measures among plasma n-3 PUFAs in early pregnancy 18. Moreover, ALA, an essential fatty acid, is converted to EPA and DHA in the human body^80^. Studies have shown that fetal height, weight, and head circumference may be correlated with neurodevelopmental outcomes^81,82^, whereas DHA and EPA are widely recognized to promote neurodevelopment in fetuses and infants ^83–85^. Besides, it has been suggested that elevated levels of maternal circulating fatty acids may lead to the development of GDM ^86^, and we have found an association between the maternal circulating fatty acids ALA and DHA with GDM in a previous study ^87^. These findings support the hypothesis that maternal high fatty acid ALA intake in early pregnancy may be associated with GDM 88 and results in early fetal overgrowth ^88,89^, but that the rate of fetal growth and development gradually slows down in mid-to-late gestation when health management of dietary fatty acid intake due to the development of GDM. These studies suggest a complex and diverse relationship between maternal fatty acid levels during pregnancy and intrauterine fetal growth and development, just as our study showed an association between maternal ALA increase in early pregnancy and the dramatically falling trajectory group.

Furthermore, we found potential sex differences in the effects of maternal fatty acids on fetal growth trajectories during pregnancy. In our study, the effects of maternal serum EDA and the remaining ten except n-3 and n-6 (especially lignoceric acid and tetracosenic acid) were more pronounced in female fetal growth. In contrast, arachidic acid was more pronounced in male fetal growth. Only a few studies from animal experiments have reported sex-specific effects of fatty acids on fetal growth ^90,91^. For example, in a study using pigs as animal models, it was shown that female fetuses have a higher availability of essential fatty acids than males and that prenatal developmental and metabolic traits are primarily determined by fetal sex and are strongly regulated by fetal genotype 91, and in another study in a rat model, it was also shown that total fatty acid content of the maternal diet and the LA: ALA ratio have a sex-specific effect on the growth of the developing fetus 92. In addition, few other studies have reported sex-specific impacts of fatty acids on fetal growth, and the results of a study from a Multi-Racial/Ethnic Birth Cohort in the US showed that SFAs with even and very long even chains were negatively associated with fetal weight and size^92^. Although this study did not discuss the sex-specificity of the effects of these fatty acids on fetal growth, but their study found an association between SFAs (such as Lignoceric acid and Arachidic acid) and fetal growth, and our findings further identified sex-specific effects between these fatty acids and fetal growth trajectory groups. Lastly, given that fetal sex affects placental lipid metabolism and may be a vital modifier of the impact of maternal metabolic health on perinatal outcomes^93^, the role of altered fetal sex should be considered in future studies. These associations suggest that maternal fatty acid status early in pregnancy may influence GDM or reflect fetal growth potential, and can be used as a predictive biomarker. Clinically, this means that a simple blood test before the 14th week of gestation could help identify pregnancies at risk of suboptimal growth patterns. Such early identification would allow for timely interventions—including closer sonographic monitoring, tailored nutritional guidance, or referral to maternal–fetal medicine specialists—thereby shifting prenatal care from reactive to proactive.

This study utilized the GBMTM framework to integrate four fetal core biometric parameters, revealing the dynamic characteristics of fetal growth. Compared to traditional methods and single-dimensional evaluation systems such as EFW, the GBMTM framework significantly enhances the comprehensiveness of growth trajectory analysis through multi-parameter joint modeling. Additionally, to validate the validity of single serum fatty acid analysis results and reduce the likelihood of chance discoveries, we analyzed the effects of individual fatty acids, n-3 and n-6 PUFAs, and a mixture of twenty fatty acids. Although some mixed analysis results did not show statistical significance, the fatty acid results from covariate-adjusted analysis and IPTW analysis were consistent, highlighting the robustness of our findings.

This study has several limitations worth noting. First, GBMTM is a semi-parametric finite mixture model, and like other trajectory analysis methods, it cannot perfectly describe the trajectory of each individual. Therefore, we encourage the use of other trajectory modeling methods and model robustness analysis to validate our results. Second, although multiple confounding factors were considered, unrecorded variables such as maternal dietary structure, use of nutritional supplements, and exposure to environmental pollutants may influence maternal fatty acid metabolism. Additionally, this study only conducted association analyses and did not validate the experimental results or explore them further. Future studies could validate the results obtained through animal experiments and explore their potential mechanisms. Finally, all participants in this study were recruited from a single medical institution in southwest China, and the specificity of regional culture, dietary habits, and genetic background may limit the external generalizability of the conclusions. Future studies should combine multi-regional cohorts, multi-omics data, and longitudinal tracking designs to further elucidate the complex association between maternal fatty acids and fetal development.

## Conclusion

In summary, we observed that elevated levels of partial fatty acids in mothers during early pregnancy, particularly LA and ALA, were associated with increased odds in the stable falling and dramatically falling trajectory groups, whereas EDA was associated with decreased odds in the stable falling trajectory group. In addition, there was a sex-specific association between changes in partial fatty acid levels and different fetal growth trajectories. Given the nonlinear relationship presented by ALA and Arachidic acid in the GAM model and the importance of fetal growth trajectories on the birth outcomes of healthy fetuses, further observational and experimental studies are needed in the future to confirm the current findings.

## Supplementary Information

Supplementary Information.

## Data Availability

The datasets used during the current study are available from the corresponding author on reasonable request.

## Abbreviations

AC: Abdominal circumference
ALA: α-Linolenic acid
Avepp: Average a posteriori probability
BPD: Biparietal diameter
BMI: Body mass index
BIC: Bayesian information criteria
CLIMB: The complex lipids in mothers and babies cohort
CI: Confidence intervals
CUDA: Chinese ultrasonographers’ association
DAG: Directed acyclic graph
DHA: Docosahexaenoic acid
EDA: Eicosadienoic acid
EPA: Eicosapentaenoic acid
FL: Femur length
GBMTM: Group-based multi-trajectory modeling
GC–MS: Gas chromatography-mass spectrometry
HC: Head circumference
IPTW: Inverse probability of treatment weights
LGA: Large-for-gestational-age
LMP: Last menstrual period
LA: Linoleic acid
MUFA: Monounsaturated fatty acids
OR: Odds ratios
PUFA: Polyunsaturated fatty acids
Qgcomp: Quantile-based g-computation
WQS: Weighted quantile sum
