# Supplementary material for: Multivariate fetal growth trajectory modeling and its association with maternal fatty acids
Source: Sci Rep. 2025 Dec 30;16:744. doi: 10.1038/s41598-025-30334-5 (PMC12780062; doi:10.1038/s41598-025-30334-5)
Supplement: Supplementary file 1 — Supplementary Information. [file 41598_2025_30334_MOESM1_ESM.docx]

**Supplemental Material**

**Multivariate fetal growth trajectory modeling and its association with maternal fatty acids**

**Supplementary Methods**

**eTable 1.** Model fit indices for separate group-based trajectory models (GBTM) of individual fetal growth parameter z-scores in the CLIMB study.

**eTable 2.** Model fit indices for 3, 4 and 5 class group-based multi-trajectory modeling (GBMTM) of four fetal growth parameter Z-scores in the CLIMB study.

**eTable 3**. Comparison of the incidence of adverse pregnancy outcomes in four fetal

**eTable 4.** Odds ratio (OR) and 95% confidence interval (CI) for each trajectory groups according to per doubling increase in prenatal fatty acids level (mg/L).

**eTable 5.** Weights for each fatty acids in quantile g-computation (qgcomp) model in CLIMB study.

**eTable 6.** Odds ratio (OR) and 95% confidence interval (CI) for each trajectory groups according to per doubling increase in prenatal FAs level (stratified by sex).

**eTable 7.** Weights for each fatty acids in quantile g-computation (qgcomp) model in CLIMB study (stratified by sex).

**eTable 8.** *P* values for departure from linearity in generalized additive models (GAM) in CLIMB study.

**eFigure 1:** The directed acyclic graph (DAG) for all potential confounders considered in the CLIMB study.

**eFigure 2:** Relationship between prenatal fatty acids level (mg/L) and trajectory groups in the generalized additive models (GAM), both the fitted and 95% confidence interval lines are presented in CLIMB study (stable falling vs. stable increasing).

**eFigure 3:** Relationship between prenatal fatty acids level (mg/L) and trajectory groups in the generalized additive models (GAM), both the fitted and 95% confidence interval lines are presented in CLIMB study (high stable increasing vs. stable increasing).

**eFigure 4:** Relationship between prenatal fatty acids level (mg/L) and trajectory groups in the generalized additive models (GAM), both the fitted and 95% confidence interval lines are presented in CLIMB study (dramatically falling vs. stable increasing).

**eTable 1.** Bayesian information criterion (BIC) and 2∆BIC for model selection in group-based trajectory modeling (GBTM) in the CLIMB study.

|  | | BIC^1^  (N=3240) | BIC^2^  (N=1080) | AIC | Entropy | 2△BIC | The proportion of each trajectory group | | | | | |
| --- | --- | --- | --- | --- | --- | --- | --- | --- | --- | --- | --- | --- |
|  |  |  |  |  |  |  | Groups | | | | | |
|  |  |  |  |  |  |  | 1 | 2 | 3 | 4 | 5 | 6 |
| BPD | | | | | | | | | | | | |
| 1 Group | 1 | -4602.96 | -4601.31 | -4593.83 |  |  | 100.00 |  |  |  |  |  |
| 2 Groups | 1 1 | -4570.56 | -4567.26 | - -4552.31 | 0.963 | 32.40 | 97.51 | 2.48 |  |  |  |  |
| 3 Groups | 2 2 2 | -4518.64 | -4512.05 | -4482.14 | 0.590 | 51.92 | 40.03 | 56.35 | 3.61 |  |  |  |
| 4 Groups* | 1 1 1 2 | -4500.99 | -4493.85 | -4461.45 | 0.718 | 17.65 | 0.59 | 68.53 | 27.13 | 3.74 |  |  |
| 5 Groups | 1 1 1 1 2 | -4507.81 | -4499.02 | -4459.14 | 0.751 | -6.82 | 1.61 | 0.59 | 68.75 | 26.22 | 2.80 |  |
| 6 Groups | 1 1 1 1 1 1 | -4539.38 | --4529.49 | -4484.63 | 0.751 | -31.57 | 0.18 | 1.74 | 68.24 | 0.23 | 26.66 | 2.92 |
| HC | | | | | | | | | | | | |
| 1 Group | 1 | -4604.75 | --4603.10 | -4595.63 |  |  | 100.00 |  |  |  |  |  |
| 2 Groups | 1 1 | -4214.06 | -4208.56 | -4183.64 | 1.000 | 277.61 | 99.09 | 0.90 |  |  |  |  |
| 3 Groups | 1 1 1 | -4260.27 | -4255.32 | -4232.80 | 1.000 | 66.88 | 0.83 | 98.98 | 0.18 |  |  |  |
| 4 Groups | 1 1 1 2 | -4171.91 | -4164.77 | -4132.37 | 0.958 | 88.35 | 0.91 | 93.93 | 4.96 | 0.18 |  |  |
| 5 Groups* | 1 1 1 1 1 | -4121.60 | -4110.63 | -4060.70 | 0.965 | -26.67 | 3.56 | 0.83 | 34.13 | 61.27 | 0.18 |  |
| 6 Groups | 1 1 1 1 1 1 | -4207.02 | -4197.13 | -4152.20 | 0.881 | -8.43 | 0.09 | 88.79 | 6.51 | 0.83 | 0.18 | 3.57 |
| AC | | | | | | | | | | | | |
| 1 Group | 1 | -4603.94 | -4602.29 | -4594.81 |  |  | 100.00 |  |  |  |  |  |
| 2 Groups | 1 1 | -4407.13 | -4403.83 | -4388.88 | 1.000 | 196.81 | 0.18 | 99.81 |  |  |  |  |
| 3 Groups | 1 2 2 | -4343.51 | -4337.46 | -4310.05 | 1.000 | 63.62 | 99.44 | 0.27 | 0.27 |  |  |  |
| 4 Groups* | 1 1 1 2 | -3969.34 | -3958.90 | -3911.55 | 0.973 | -9.24 | 44.26 | 55.08 | 0.46 | 0.18 |  |  |
| 5 Groups | 1 1 1 1 1 | -4298.16 | -4289.37 | -4249.49 | 0.971 | -14.60 | 0.46 | 96.26 | 0.18 | 2.99 | 0.09 |  |
| 6 Groups | 1 1 1 2 2 2 | -4251.30 | -4239.78 | -4187.44 | 0.835 | 116.03 | 0.27 | 78.55 | 16.66 | 0.94 | 0.09 | 3.40 |
| FL | | | | | | | | | | | | |
| 1 Group | 1 | -4602.19 | -4600.54 | -4593.07 |  |  | 100.00 |  |  |  |  |  |
| 2 Groups | 1 1 | -4553.08 | -4549.79 | -4534.83 | 0.957 | 49.11 | 96.91 | 3.08 |  |  |  |  |
| 3 Groups | 1 1 1 | -4510.58 | -4505.64 | -4483.20 | 0.971 | 42.51 | 96.55 | 0.09 | 3.35 |  |  |  |
| 4 Groups | 1 1 1 2 | -4493.05 | -4485.91 | -4453.51 | 0.959 | 17.53 | 1.62 | 94.45 | 0.27 | 3.65 |  |  |
| 5 Groups | 1 1 1 1 1 | -4496.39 | -4488.15 | -4450.77 | 0.955 | -3.34 | 1.43 | 1.07 | 93.85 | 0.09 | 3.54 |  |
| 6 Groups* | 2 1 1 1 1 1 | -4487.26 | -4476.82 | -4429.47 | 0.791 | 9.13 | 4.18 | 9.61 | 23.00 | 34.44 | 20.79 | 7.98 |

Note: * represents the smallest absolute value of BIC; BIC^1^ with N represents three times one fetal growth parameter z-scores 3×1080=3240; BIC^2^ with N represents 1080 participates one fetal growth parameter Z-scores.

**eTable 2.** Model fit indices for 3, 4 and 5 class group-based multi-trajectory modeling (GBMTM) of four fetal growth parameter z-scores in the CLIMB study.

|  | | | BIC  (N=12960) | AIC |  | The proportion of each trajectory group (AvePP) | | | | | | | | |  |  |  |  |
| --- | --- | --- | --- | --- | --- | --- | --- | --- | --- | --- | --- | --- | --- | --- | --- | --- | --- | --- |
|  |  | BPD  HC  AC  FL |  |  |  | 1 | 2 | 3 | | 4 | | 5 | | |  |  |  |  |
| 3 trajectories |  | | | | | | | | | | | | | |  |  |  |  |
|  | Purely quadratic | 2 2 2 2 2 2 2 2 2  2 2 2 | -17233.06 | -17076.20 |  | 43.42 (0.90) | 47.04 (0.89) | | 9.53(0.93) | |  | |  | | |  |  |  |
|  | Polynomials suggested by GBTM | 2 2 2 1 1 1 1 2 2  1 1 1 | -17256.74 | -17126.03 |  | 43.25 (0.90) | 46.51 (0.90) | | 10.22 (0.95) | |  | |  | | |  |  |  |
|  | Purely Linear | 1 1 1 1 1 1 1 1 1  1 1 1 | -17288.67 | -17176.62 |  | 41.89 (0.91) | 46.14 (0.91) | | 11.97 (0.96) | |  | |  | | |  |  |  |
| 4 trajectories |  | | | | | | | | | | | | | |  |  |  |  |
|  | Purely quadratic | 2 2 2 2 2 2 2 2 2 2 2 2  2 2 2 2 | -17117.26 | -16911.85 |  | 55.65 (0.90) | 15.66 (0.85) | | 19.77 (0.86) | | 8.91 (0.92) | |  | | |  |  |  |
|  | Polynomials suggested by GBTM ^#^ | 1 1 1 2 1 1 1 2 1 1 1 2  1 1 1 2 | -17110.42 | -16949.82 |  | 19.32 (0.86) | 56.28 (0.90) | | 15.39 (0.87) | | 8.99 (0.95) | |  | | |  |  |  |
|  | Purely Linear | 1 1 1 1 1 1 1 1 1 1 1 1  1 1 1 1 | -17168.96 | -17023.31 |  | 55.77 (0.90) | 13.95 (0.84) | | 18.62 (0.86) | | 11.65 (0.92) | | |  | | |  |  |
|  | | | | | | | | | | | | | | |  |  |  |  |
| 5 trajectories | Purely quadratic* | 2 2 2 2 2 2 2 2 2 2 2 2 2 2 2  2 2 2 2 2 | -16930.78 | -16676.81 |  | 18.52 (0.88) | 49.40 (0.89) | | 15.11 (0.88) | | 13.47 (0.88) | | | 3.47 (0.95) | | | |  |
|  | Polynomials suggested by GBTM | 1 1 1 1 2 1 1 1 1 1 1 1 1 1 1  1 1 1 1 1 | -16972.36 | -16789.36 |  | 17.34 (0.87) | 45.37 (0.86) | | 14.36 (0.86) | | 18.26 (0.85) | | | 4.65 (0.95) | | | | |
|  | Purely linear | 1 1 1 1 1 1 1 1 1 1 1 1 1 1 1  1 1 1 1 1 | -17014.44 | -16835.17 |  | 17.34 (0.86) | 46.6 9(0.87) | | 14.17 (0.86) | | 17.63 (0.85) | | | 4.15 (0.95) | | | | |

Note**:** * represents the smallest absolute value of BIC；N represents three times four fetal growth parameter Z-scores 3×4×1080=12960; ^#^ represents adequacy of model fit in GBMTM, a lower absolute BIC value, AvePPs ≥0.7, and the proportion of each trajectory group ≥ 5%.

**eTable 3.** Comparison of the incidence of adverse pregnancy outcomes in four fetal growth trajectory groups.

| Adverse pregnancy outcomes | stable  falling  (n=116) | | stable increasing  (n=394) | high stable increasing  (n=92) | | | dramatically falling  (n=53) | | *p*-value |  |
| --- | --- | --- | --- | --- | --- | --- | --- | --- | --- | --- |
| Gestational diabetes mellitus | 35 (30.2) | 104 (26.4) | | | 23 (25.0) | 14 (26.4) | | 0.836 | | |
| Gestational hypertension | 1 (0.9) | 14 (3.6) | | | 1 (1.1) | 2 (3.8) | | 0.301 | | |
| Prematurity | 1 (0.9) | 12 (3.0) | | | 0 (0.0) | 1 (1.9) | | 0.214 | | |
| Preeclampsia | 0 (0.0) | 5 (1.3) | | | 0 (0.0) | 1 (1.9) | | 0.389 | | |
| Fetal distress | 0 (0.0) | 7 (1.8) | | | 2 (2.2) | 1 (1.9) | | 0.519 | | |
| Cesarean delivery | 45 (38.8) | 151 (38.3) | | | 38 (41.3) | 20 (37.7) | | 0.959 | | |
| Premature rupture of membranes | 30 (25.9) | 97 (24.6) | | | 14 (15.2) ^b^ | 19 (35.8) ^a^ | | **0.044*** | | |
| Chorioamnionitis | 1 (0.9) | 7 (1.8) | | | 0 (0.0) | 2 (3.8) | | 0.294 | | |
| Anemia of pregnancy | 2 (1.7) | 13 (3.3) | | | 2 (2.2) | 2 (3.8) | | 0.775 | | |
| Hypothyroidism | 9 (7.8) | 17 (4.3) | | | 2 (2.2) | 3 (5.7) | | 0.267 | | |
| Abnormality of fetal position | 6 (5.2) | 9 (2.3) | | | 4 (4.3) | 1 (1.9) | | 0.346 | | |
| Nuchal cord | 27 (23.3) | 62 (15.7) | | | 17 (18.5) | 14 (26.4) | | 0.112 | | |
| Placental implantation | 9 (7.8) | 27 (6.9) ^b^ | | | 8 (8.7) | 11 (20.8) ^a^ | | **0.008*** | | |
| Oligohydramnios | 3 (2.6) | 9 (2.3) | | | 2 (2.2) | 0 (0.0) | | 0.727 | | |
| Small for Gestational age infant | 15 (12.9) | 26 (6.6) | | | 4 (4.3) | 5 (9.4) | | 0.076 | | |
| Large for Gestational Age | 3 (2.6) | 32 (8.1) ^b^ | | | 21 (22.8) ^a^ | 6 (11.3) | | **<0.001*** | | |
| Low Birth Weight | 2 (1.7) | 14 (3.6) | | | 1 (1.1) | 1 (1.9) | | 0.474 | | |
| Macrosomia | 2 (1.7) | 19 (4.8) ^b^ | | | 15 (16.3) ^a^ | 4 (7.5) | | **<0.001*** | | |

Note**:** *p*-values were calculated using Chi-square tests or Fisher’s exact tests, as appropriate, **p* < 0.05 indicates statistical significance, Data are presented as n (%); “a” Indicates that the group showed statistically significant differences (*p* <0.05) compared to at least one other group in post-hoc pairwise comparisons; “b” Indicates that the reference group.

**eTable 4.** Odds ratio (OR) and 95% confidence interval (CI) for each trajectory groups according to per doubling increase in prenatal fatty acids level (mg/L).

| Fatty acids | Unadjusted | | | Adjusted | | | | | | | IPTW-adjusted | | | | | | | | | | | | |  |  |  |  |  |
| --- | --- | --- | --- | --- | --- | --- | --- | --- | --- | --- | --- | --- | --- | --- | --- | --- | --- | --- | --- | --- | --- | --- | --- | --- | --- | --- | --- | --- |
|  | **stable falling vs.**  **stable increasing (n=116 vs. n=394) 1 vs.2** | | | | **stable falling vs.**  **stable increasing (n=116 vs. n=394) 1 vs.2** | | | | | | | | | **stable falling vs.**  **stable increasing**  **(n=116 vs. n=394) 1 vs.2** | | | | | | | | | | | | | | |
| Docosahexaenoic acid | | | 0.997(0.982~1.013) | | | 0.996(0.980~1.012) | | | | | | | 0.997(0.981~1.012) | | | | | | | | | | | |  |  |  |  |
| Eicosapentaenoic acid | | | 1.009(0.970~1.050) | | | 1.008(0.969~1.049) | | | | | | | 1.006(0.966~1.047) | | | | | | | | | | | |  |  |  |  |
| α-linolenic acid | | | 0.979(0.950~1.008) | | | 0.979(0.950~1.009) | | | | | | | 0.979(0.950~1.009) | | | | | | | | | | | |  |  |  |  |
| Docosapentenoic acid | | | 0.851(0.705~1.027) | | | 0.853(0.703~1.034) | | | | | | | 0.865(0.713~1.049) | | | | | | | | | | | |  |  |  |  |
| n-3 PUFAs | | | 0.918(0.715~1.179) | | | 0.911(0.708~1.172) | | | | | | | 0.916(0.711~1.180) | | | | | | | | | | | |  |  |  |  |
| γ-linolenic acid | | | 0.970(0.904~1.040) | | | 0.973(0.906~1.045) | | | | | | | 0.982(0.915~1.052) | | | | | | | | | | | |  |  |  |  |
| Arachidonic acid | | | 1.001(0.992~1.009) | | | 1.001(0.992~1.009) | | | | | | | 1.001(0.993~1.010) | | | | | | | | | | | |  |  |  |  |
| Docosatetraenoic acid | | | 1.034(0.799~1.337) | | | 1.029(0.791~1.338) | | | | | | | 1.031(0.794~1.339) | | | | | | | | | | | |  |  |  |  |
| Eicosadienoic acid | | | 0.858(0.729~1.009) | | | **0.841(0.712~0.993)*** | | | | | | | **0.845(0.716~0.998)*** | | | | | | | | | | | |  |  |  |  |
| Eicosatrienoic acid | | | 0.996(0.981~1.012) | | | 0.998(0.982~1.015) | | | | | | | 0.998(0.982~1.014) | | | | | | | | | | | |  |  |  |  |
| Linoleic acid | | | **1.004(****1.000~1.007)*** | | | **1.005(1.001~1.008)*** | | | | | | | **1.004(1.001~1.008)*** | | | | | | | | | | | |  |  |  |  |
| n-6 PUFAs | | | 0.896(0.692~1.159) | | | 0.915(0.704~1.190) | | | | | | | 0.921(0.708~1.199) | | | | | | | | | | | |  |  |  |  |
| Arachidic acid | | | 0.984(0.705~1.374) | | | 0.989(0.703~1.389) | | | | | | | 0.958(0.682~1.344) | | | | | | | | | | | |  |  |  |  |
| Docosanoic acid | | | 1.008(0.905~1.123) | | | 1.002(0.899~1.118) | | | | | | | 1.000(0.897~1.115) | | | | | | | | | | | |  |  |  |  |
| Eicosaenoic acid | | | 1.098(0.970~1.243) | | | 1.087(0.959~1.233) | | | | | | | 1.083(0.954~1.230) | | | | | | | | | | | |  |  |  |  |
| Hexadecenoic acid | | | 1.029(0.990~1.070) | | | 1.031(0.991~1.072) | | | | | | | 1.028(0.988~1.069) | | | | | | | | | | | |  |  |  |  |
| Hexadecanoic acid | | | 0.997(0.990~1.003) | | | 0.996(0.989~1.002) | | | | | | | 0.996(0.989~1.002) | | | | | | | | | | | |  |  |  |  |
| Lignoceric acid | | | 1.163(0.964~1.403) | | | 1.163(0.958~1.412) | | | | | | | 1.146(0.943~1.393) | | | | | | | | | | | |  |  |  |  |
| Octadecanoic acid | | | 1.002(0.986~1.017) | | | 1.000(0.984~1.016) | | | | | | | 1.001(0.986~1.017) | | | | | | | | | | | |  |  |  |  |
| Octadecenoic acid | | | 1.001(0.995~1.007) | | | 1.002(0.996~1.007) | | | | | | | 1.002(0.996~1.008) | | | | | | | | | | | |  |  |  |  |
| Tetracosenic acid | | | 1.001(0.970~1.032) | | | 1.003(0.972~1.035) | | | | | | | 1.003(0.973~1.035) | | | | | | | | | | | |  |  |  |  |
| Tetradecanoic acid | | | 1.021(0.967~1.078) | | | 1.025(0.969~1.085) | | | | | | | 1.023(0.968~1.082) | | | | | | | | | | | |  |  |  |  |
| other fatty acid mixtures | | | | 0.805(0.598~1.084) | | | | 0.812(0.601~1.097) | | | | | 0.815(0.603~1.101) | | | | | | | | | | | | | |  |  |
| ∑fatty acid mixtures | | | 0.789(0.559~1.114) | | | | 0.805(0.567~1.143) | | | | | 0.809(0.569~1.148) | | | | | | | | | | | | | |  |  |  |
|  | | **high stable increasing vs. stable increasing (n=92 vs. n=394)3 vs.2** | | | | | | | **high stable increasing vs. stable increasing (n=92 vs. n=394)3 vs.2** | | | | | | | **high stable increasing vs. stable increasing**  **(n=92 vs. n=394)3 vs.2** | | | | | | | | | | |  |  |
| Docosahexaenoic acid | | | 0.999(0.982~1.016) | | | | 1.000(0.983~1.017) | | | | | | | | 1.001(0.985~1.019) | | | |  |  |  |  |  |  |  |  |  |  |
| Eicosapentaenoic acid | | | 1.001(0.958~1.047) | | | | 1.003(0.958~1.049) | | | | | | | | 1.001(0.958~1.047) | | | |  |  |  |  |  |  |  |  |  |  |
| α-linolenic acid | | | 1.010(0.980~1.040) | | | | 1.010(0.980~1.040) | | | | | | | | 1.010(0.980~1.040) | | | |  |  |  |  |  |  |  |  |  |  |
| Docosapentenoic acid | | | 0.984(0.807~1.199) | | | | 0.955(0.780~1.168) | | | | | | | | 0.971(0.796~1.185) | | | |  |  |  |  |  |  |  |  |  |  |
| n-3 PUFAs | | | 1.035(0.788~1.360) | | | | 1.038(0.786~1.372) | | | | | | | | 1.032 (0.779~1.366) | | | | |  |  |  |  |  |  |  |  |  |
| γ-linolenic acid | | | 0.927(0.847~1.014) | | | | 0.925(0.844~1.014) | | | | | | | | 0.922(0.840~1.011) | | | | | |  |  |  |  |  |  |  |  |
| Arachidonic acid | | | 0.994(0.985~1.004) | | | | | 0.994(0.984~1.004) | | | | | | | 0.994(0.984~1.003) | | | | | |  |  |  |  |  |  |  |  |
| Docosatetraenoic acid | | | 1.134(0.855~1.504) | | | | | 1.150(0.864~1.532) | | | | | | | 1.191(0.893~1.588) | | | | | |  |  |  |  |  |  |  |  |
| Eicosadienoic acid | | | 0.910(0.760~1.089) | | | | | 0.911(0.760~1.093) | | | | | | | 0.910(0.760~1.089) | | | | | | | |  |  |  |  |  |  |
| Eicosatrienoic acid | | | 1.001(0.983~1.019) | | | | | 0.999(0.981~1.018) | | | | | | | 0.999(0.980~1.017) | | | | | | | |  |  |  |  |  |  |
| Linoleic acid | | | 1.003(0.999~1.007) | | | | | 1.003(0.999~1.007) | | | | | | | 1.003(0.999~1.007) | | | | | | | |  |  |  |  |  |  |
| n-6 PUFAs | | | 0.995(0.749~1.322) | | | | | 1.007(0.753~1.346) | | | | | | | 1.003(0.749~1.342) | | | | | | | |  |  |  |  |  |  |
| Arachidic acid | | | 0.743(0.514~1.073) | | | | | 0.764(0.525~1.112) | | | | | | | 0.772(0.532~1.119) | | | | | | | |  |  |  |  |  |  |
| Docosanoic acid | | | 1.028(0.915~1.156) | | | | | 1.033(0.915~1.165) | | | | | | | 1.025(0.910~1.154) | | | | | | | |  |  |  |  |  |  |
| Eicosaenoic acid | | | 1.053(0.915~1.212) | | | | | 1.063(0.921~1.226) | | | | | | | 1.074(0.937~1.232) | | | | | | | |  |  |  |  |  |  |
| Hexadecenoic acid | | | 1.014(0.969~1.062) | | | | | 1.013(0.967~1.062) | | | | | | | 1.014(0.968~1.063) | | | | | | | |  |  |  |  |  |  |
| Hexadecanoic acid | | | 0.997(0.989~1.004) | | | | | 0.997(0.989~1.004) | | | | | | | 0.997(0.989~1.004) | | | | | | | |  |  |  |  |  |  |
| Lignoceric acid | | | 1.059(0.868~1.293) | | | | | 1.084(0.884~1.330) | | | | | | | 1.079(0.882~1.321) | | | | | | | |  |  |  |  |  |  |
| Octadecanoic acid | | | **1.017(1.001~1.034)*** | | | | | | | **1.017(1.000~1.034)*** | | | | | | | 1.016(0.999~1.033) | | | |  |  |  |  |  |  |  |  |
| Octadecenoic acid | | | 0.998(0.991~1.004) | | | | | 0.997(0.990~1.003) | | | | | | | 0.997(0.991~1.003) | | | | | | | |  |  |  |  |  |  |
| Tetracosenic acid | | | 1.012(0.980~1.046) | | | | | 1.013(0.979~1.048) | | | | | | | 1.011(0.977~1.045) | | | | | | | |  |  |  |  |  |  |
| Tetradecanoic acid | | | 0.983(0.920~1.052) | | | | | 0.984(0.918~1.055) | | | | | | | 0.982(0.917~1.052) | | | | | | | |  |  |  |  |  |  |
| other fatty acid mixtures | | | | 1.059(0.769~1.460) | | | | | 1.052(0.756~1.464) | | | | | | | 1.050(0.754~1.461) | | | | | | |  |  |  |  |  |  |
| ∑fatty acid mixtures | | | 1.027(0.708~1.488) | | | | | 1.026 (0.700~1.504) | | | | | | | 1.023 (0.697~1.499) | | | | | | |  |  |  |  |  |  |  |
|  | | **dramatically falling vs. stable increasing**  **(n=53 vs. n=394)4 vs.2** | | | | | | | **dramatically falling vs. stable increasing**  **(n=53 vs. n=394)4 vs.2** | | | | | | | | | **dramatically falling vs. stable increasing**  **(n=53 vs. n=394)4 vs.2** | | | | | | | | | |  |
| Docosahexaenoic acid | | | 1.009(0.986~1.032) | | | | | 1.007(0.984~1.031) | | | | | | | 1.008(0.986~1.031) | | | | | | | |  |  |  |  |  |  |
| Eicosapentaenoic acid | | | 0.945(0.873~1.023) | | | | | 0.948(0.874~1.028) | | | | | | | 0.953(0.882~1.030) | | | | | | | |  |  |  |  |  |  |
| α-linolenic acid | | | **1.045(1.003~1.088)*** | | | | | **1.046(1.004~1.089)*** | | | | | | | **1.042(1.001~1.085)*** | | | | | | | |  |  |  |  |  |  |
| Docosapentenoic acid | | | 0.911(0.710~1.168) | | | | | 0.910(0.700~1.183) | | | | | | | 0.925(0.714~1.199) | | | | | | | |  |  |  |  |  |  |
| n-3 PUFAs | | | 0.899(0.631~1.279) | | | | | 0.902(0.633~ 1.284) | | | | | | | 0.903(0.633~1.287) | | | | | | | |  |  |  |  |  |  |
| γ-linolenic acid | | | 0.891(0.780~1.019) | | | | | 0.872(0.759~1.001) | | | | | | | 0.878(0.766~1.006) | | | | | | | |  |  |  |  |  |  |
| Arachidonic acid | | | 1.006(0.993~1.018) | | | | | 1.007(0.994~1.020) | | | | | | | 1.006(0.994~1.019) | | | | | | | |  |  |  |  |  |  |
| Docosatetraenoic acid | | | 0.832(0.578~1.197) | | | | | 0.847(0.582~1.231) | | | | | | | 0.853(0.589~1.236) | | | | | | | |  |  |  |  |  |  |
| Eicosadienoic acid | | | 1.020(0.811~1.283) | | | | | 0.996(0.784~1.264) | | | | | | | 0.982(0.774~1.246) | | | | | | | |  |  |  |  |  |  |
| Eicosatrienoic acid | | | 1.003(0.981~1.026) | | | | | 1.007(0.984~1.030) | | | | | | | 1.007(0.985~1.030) | | | | | | | |  |  |  |  |  |  |
| Linoleic acid | | | 1.000(0.995~1.005) | | | | | 1.001(0.996~1.006) | | | | | | | 1.002(0.996~1.007) | | | | | | | |  |  |  |  |  |  |
| n-6 PUFAs | | | 0.800(0.556~1.151) | | | | | 0.806(0.558~1.165) | | | | | | | 0.807(0.558~1.167) | | | | | | | |  |  |  |  |  |  |
| Arachidic acid | | | 1.287(0.806~2.055) | | | | | 1.299(0.806~2.094) | | | | | | | 1.226(0.762~1.974) | | | | | | | |  |  |  |  |  |  |
| Docosanoic acid | | | 0.942(0.788~1.126) | | | | | 0.928(0.773~1.113) | | | | | | | 0.927(0.774~1.110) | | | | | | | |  |  |  |  |  |  |
| Eicosaenoic acid | | | 0.784(0.554~1.109) | | | | | 0.779(0.546~1.112) | | | | | | | 0.807(0.568~1.146) | | | | | | | |  |  |  |  |  |  |
| Hexadecenoic acid | | | 0.993(0.931~1.058) | | | | | 0.989(0.927~1.056) | | | | | | | 1.003(0.941~1.068) | | | | | | | |  |  |  |  |  |  |
| Hexadecanoic acid | | | 0.999(0.989~1.004) | | | | | 0.998(0.989~1.008) | | | | | | | 0.998(0.989~1.008) | | | | | | | |  |  |  |  |  |  |
| Lignoceric acid | | | 1.119(0.861~1.453) | | | | | 1.117(0.849~1.469) | | | | | | | 1.105(0.842~1.450) | | | | | | | |  |  |  |  |  |  |
| Octadecanoic acid | | | 1.005(0.983~1.028) | | | | | 1.004(0.981~1.027) | | | | | | | 1.003(0.981~1.026) | | | | | | | |  |  |  |  |  |  |
| Octadecenoic acid | | | 0.996(0.987~1.006) | | | | | 0.996(0.987~1.006) | | | | | | | 0.995(0.986~1.005) | | | | | | | |  |  |  |  |  |  |
| Tetracosenic acid | | | 0.985(0.937~1.036) | | | | | 0.985(0.935~1.038) | | | | | | | 0.985(0.935~1.037) | | | | | | | |  |  |  |  |  |  |
| Tetradecanoic acid | | | 1.055(0.981~1.135) | | | | | 1.069(0.992~1.152) | | | | | | | 1.058(0.981~1.142) | | | | | | | |  |  |  |  |  |  |
| other fatty acid mixtures | | | | 1.017(0.676~1.530) | | | | | 1.044(0.691~1.576) | | | | | | | 1.046(0.693~1.579) | | | | | | | |  |  |  |  |  |
| ∑fatty acid mixtures | | | 0.852(0.522~1.391) | | | | | 0.858(0.523~1.409) | | | | | | | 0.859(0.523~1.412) | | | | | | | |  |  |  |  |  |  |

Note**:** Values are presented as odds ratios (95% confidence intervals), the adjusted model included covariates such as socio-economic status, maternal age, BMI, fetal sex, smoking/drinking and han ethnic groups. IPTW-adjusted models account for potential confounding using inverse probability of treatment weighting based on propensity scores, **p* < 0.05, indicating statistical significance.

**eTable 5.** Weights for each fatty acids in quantile g-computation (Qgcomp) model in CLIMB study.

| Fatty acids | | Unadjusted | | Adjusted | | IPTW-adjusted | |
| --- | --- | --- | --- | --- | --- | --- | --- |
|  |  | **stable falling vs. stable increasing**  **(n=116vs. n=394) 1 vs.2** | | **stable falling vs. stable increasing**  **(n=116vs. n=394) 1 vs.2** | | **stable falling vs. stable increasing**  **(n=116 vs. n=394) 1 vs.2** | |
| Docosahexaenoic acid | | 0.044 | | 0.025 | | 0.030 | |
| Eicosapentaenoic acid | | -0.007 | | 0.003 | | 0.001 | |
| α-linolenic acid | | 0.132 | | 0.110 | | 0.109 | |
| Docosapentenoic acid | | 0.067 | | 0.070 | | 0.072 | |
| γ-linolenic acid | | -0.028 | | 0.008 | | 0.009 | |
| Arachidonic acid | | -0.063 | | -0.066 | | -0.064 | |
| Docosatetraenoic acid | | -0.014 | | -0.030 | | -0.030 | |
| Eicosadienoic acid | | -0.027 | | -0.026 | | -0.027 | |
| Eicosatrienoic acid | | -0.062 | | -0.046 | | -0.042 | |
| Linoleic acid | | -0.012 | | 0.043 | | 0.045 | |
| Arachidic acid | | -0.042 | | -0.041 | | -0.042 | |
| Docosanoic acid | | -0.202 | | 0.234 | | -0.235 | |
| Eicosaenoic acid | | -0.371 | | -0.368 | | -0.368 | |
| Hexadecenoic acid | | 0.190 | | 0.194 | | 0.190 | |
| Hexadecanoic acid | | 0.184 | | 0.121 | | 0.117 | |
| Lignoceric acid | | -0.172 | | -0.188 | | -0.192 | |
| Octadecanoic acid | | 0.215 | | 0.219 | | 0.220 | |
| Octadecenoic acid | | 0.008 | | 0.012 | | 0.014 | |
| Tetracosenic acid | | 0.160 | | 0.181 | | 0.178 | |
| Tetradecanoic acid | | -0.002 | | 0.014 | | 0.014 | |
|  | **high stable increasing vs. stable increasing**  **(n=92 vs. n=394)3 vs.2** | | **high stable increasing vs. stable increasing**  **(n=92 vs. n=394)3 vs.2** | | **high stable increasing vs. stable increasing**  **(n=92 vs. n=394)3 vs.2** | |  |
| Docosahexaenoic acid | | -0.061 | | -0.055 | | -0.057 | |
| Eicosapentaenoic acid | | 0.038 | | 0.040 | | 0.040 | |
| α-linolenic acid | | 0.069 | | 0.080 | | 0.078 | |
| Docosapentenoic acid | | -0.108 | | -0.107 | | -0.113 | |
| γ-linolenic acid | | -0.112 | | -0.097 | | -0.095 | |
| Arachidonic acid | | -0.066 | | -0.085 | | -0.085 | |
| Docosatetraenoic acid | | 0.049 | | 0.072 | | 0.069 | |
| Eicosadienoic acid | | -0.072 | | -0.053 | | -0.050 | |
| Eicosatrienoic acid | | -0.010 | | -0.026 | | -0.029 | |
| Linoleic acid | | 0.143 | | 0.144 | | 0.144 | |
| Arachidic acid | | -0.140 | | -0.143 | | -0.138 | |
| Docosanoic acid | | -0.006 | | -0.009 | | -0.011 | |
| Eicosaenoic acid | | -0.176 | | -0.176 | | -0.176 | |
| Hexadecenoic acid | | -0.079 | | -0.086 | | -0.083 | |
| Hexadecanoic acid | | -0.169 | | -0.164 | | -0.164 | |
| Lignoceric acid | | 0.151 | | 0.153 | | 0.156 | |
| Octadecanoic acid | | 0.198 | | 0.200 | | 0.199 | |
| Octadecenoic acid | | 0.102 | | 0.107 | | 0.105 | |
| Tetracosenic acid | | 0.164 | | 0.160 | | 0.161 | |
| Tetradecanoic acid | | 0.086 | | 0.043 | | 0.047 | |
|  | | **dramatically falling vs. stable increasing**  **(n=53 vs. n=394)4 vs.2** | | **dramatically falling vs. stable increasing**  **(n=53 vs. n=394)4 vs.2** | | **dramatically falling vs. stable increasing**  **(n=53 vs. n=394)4 vs.2** | |
| Docosahexaenoic acid | | 0.092 | | 0.091 | | 0.092 | |
| Eicosapentaenoic acid | | -0.098 | | -0.108 | | -0.108 | |
| α-linolenic acid | | 0.271 | | 0.277 | | 0.276 | |
| Docosapentenoic acid | | 0.022 | | 0.018 | | 0.019 | |
| γ-linolenic acid | | -0.064 | | -0.070 | | -0.070 | |
| Arachidonic acid | | -0.085 | | -0.086 | | -0.086 | |
| Docosatetraenoic acid | | -0.041 | | -0.053 | | -0.052 | |
| Eicosadienoic acid | | 0.044 | | 0.031 | | 0.031 | |
| Eicosatrienoic acid | | -0.014 | | -0.026 | | 0.007 | |
| Linoleic acid | | -0.202 | | -0.190 | | -0.190 | |
| Arachidic acid | | -0.004 | | 0.009 | | 0.008 | |
| Docosanoic acid | | -0.154 | | -0.150 | | -0.150 | |
| Eicosaenoic acid | | -0.066 | | -0.081 | | -0.081 | |
| Hexadecenoic acid | | -0.008 | | -0.014 | | -0.014 | |
| Hexadecanoic acid | | 0.264 | | 0.260 | | 0.259 | |
| Lignoceric acid | | 0.216 | | 0.228 | | 0.227 | |
| Octadecanoic acid | | 0.089 | | 0.078 | | 0.078 | |
| Octadecenoic acid | | -0.239 | | -0.240 | | -0.240 | |
| Tetracosenic acid | | 0.003 | | 0.003 | | 0.002 | |
| Tetradecanoic acid | | -0.026 | | -0.008 | | -0.008 | |

Note**:** Qgcomp was used to estimate the joint effect of multiple fatty acids on fetal growth trajectories; weights represent the estimated contribution of each fatty acid to the overall effect; adjustment models included covariates such as socio-economic status, maternal age, BMI, fetal sex, smoking/drinking and han ethnic groups; the IPTW adjustment model used propensity-score-based inverse probability-of-treatment weighting to account for potential confounders; Positive weighting indicates a positive association with the outcome and negative weighting indicates a negative association with the outcome.

**eTable 6.** Odds ratio (OR) and 95% confidence interval (CI) for each trajectory groups according to per doubling increase in prenatal fatty acids level (stratified by sex).

| Fatty acids | | Unadjusted | | | | | | | | | | | | | | | |  | | | | | | | | | | | | | | | | | | | Adjusted | | | | | | | | | | | | | | | | | | | | | |  | | | | | | | | | | | | | | | | | | | | | | | | | | IPTW-adjusted | | | | | | | | | | | | | | | | | | | | | |  | | | | | | | | | | | | | | | |  |  |  |  |  |  |  |  |  |  |  |
| --- | --- | --- | --- | --- | --- | --- | --- | --- | --- | --- | --- | --- | --- | --- | --- | --- | --- | --- | --- | --- | --- | --- | --- | --- | --- | --- | --- | --- | --- | --- | --- | --- | --- | --- | --- | --- | --- | --- | --- | --- | --- | --- | --- | --- | --- | --- | --- | --- | --- | --- | --- | --- | --- | --- | --- | --- | --- | --- | --- | --- | --- | --- | --- | --- | --- | --- | --- | --- | --- | --- | --- | --- | --- | --- | --- | --- | --- | --- | --- | --- | --- | --- | --- | --- | --- | --- | --- | --- | --- | --- | --- | --- | --- | --- | --- | --- | --- | --- | --- | --- | --- | --- | --- | --- | --- | --- | --- | --- | --- | --- | --- | --- | --- | --- | --- | --- | --- | --- | --- | --- | --- | --- | --- | --- | --- | --- | --- | --- | --- | --- | --- | --- | --- |
|  |  | Male | | | | | | | | Female | | | | | | | | | | | *p* for  interaction | | | | | | | | | | | | | | | | | | Male | | | | | | | | | Female | | | | | | | | | | | | |  | | | | | | | | | | | | | | | | | | | | | | | | | Male | | | | | | | | | | | Female | | | | | | | | | | | *p* for interaction | | | | | | | | |  |  |  |  |  |  |  |  |  |  |  |  |  |  |  |  |  |
|  |  | **stable falling vs. stable increasing (n=45 vs. n=207)**  **1 vs.2** | | | | | | | | **stable falling vs. stable increasing (n=71 vs. n=187)**  **1 vs.2** | | | | | | | | | | |  |  |  |  |  |  |  |  |  |  |  |  |  |  |  |  |  |  | **stable falling vs. stable increasing (n=45 vs. n=207) 1 vs.2** | | | | | | | | | **stable falling vs. stable increasing (n=71 vs. n=187)**  **1 vs.2** | | | | | | | | | | | | | *p* for interaction | | | | | | | | | | | | | | | | | | | | | | | | | **stable falling vs. stable increasing (n=45 vs. n=207)**  **1 vs.2** | | | | | | | | | | | **stable falling vs. stable increasing (n=71 vs. n=187)**  **1 vs.2** | | | | | | | | | | |  |  |  |  |  |  |  |  |  |  |  |  |  |  |  |  |  |  |  |  |  |  |  |  |  |  |
| Docosahexaenoic acid | | | | | 1.001(0.976~1.027) | | | | | | | | | 0.981(0.958~1.004) | | | | | | | | 0.265 | | | | | | | | | | | | | | | | | | | | 0.999(0.973~1.026) | | | | | | | | 0.979(0.955~1.003) | | | | | | | | | | | | 0.341 | | | | | | | | | | | | | | | | | | | | 1.001(0.975~1.028) | | | | | | | | | 0.979(0.955~1.003) | | | | | | | | | | | 0.287 | | | | | | | | | | | | | | | | | | | | | |  |  |  |  |  |  |  |  |  |  |
| Eicosapentaenoic acid | | 0.958(0.884~1.038) | | | | | | | | 1.066(1.000~1.137) | | | | | | | | | | 0.038 | | | | | | | | | | | | | | | | | | | 0.958(0.885~1.037) | | | | | | | | | 1.056(0.987~1.130) | | | | | | | | | | | | | 0.054 | | | | | | | | | | | | | | | | | | 0.959(0.887~1.036) | | | | | | | | | | 1.055(0.986~1.129) | | | | | | | | | | | | 0.681 | | | | | | | | | | | | | | | | | | | | | |  |  |  |  |  |  |  |  |  |  |  |
| α-linolenic acid | | 0.982(0.937~1.029) | | | | | | | | 1.000(0.953~1.049) | | | | | | | | | | 0.624 | | | | | | | | | | | | | | | | | | | 0.983(0.937~1.030) | | | | | | | | | 0.999(0.950~1.050) | | | | | | | | | | | | | 0.644 | | | | | | | | | | | | | | | | | | 0.986(0.939~1.034) | | | | | | | | | | 0.998(0.949~1.050) | | | | | | | | | | | | 0.690 | | | | | | | | | | | | | | | | | | | | | |  |  |  |  |  |  |  |  |  |  |  |
| Docosapentenoic acid | | 1.002(0.750~1.338) | | | | | | | | 0.683(0.509~0.915) | | | | | | | | | | 0.065 | | | | | | | | | | | | | | | | | | | 0.987(0.725~1.343) | | | | | | | | | 0.707(0.522~0.958) | | | | | | | | | | | | | 0.111 | | | | | | | | | | | | | | | | | | 0.974(0.714~1.328) | | | | | | | | | | 0.720(0.533~0.973) | | | | | | | | | | | | 0.153 | | | | | | | | | | | | | | | | | | | | | |  |  |  |  |  |  |  |  |  |  |  |
| n-3 PUFAs | | 1.009(0.662~1.537) | | | | | | | | 0.867(0.616~1.222) | | | | | | | | | | | / | | | | | | | | | | | | | | | | | | | 1.029(0.673~ 1.573) | | | | | | | | | | | | | 0.821(0.572~1.177) | | | | | | | | | | | / | | | | | | | | | | | | | | | 1.051 (0.684~1.614) | | | | | | | | | | | | | 0.818 (0.570~1.173) | | | | | | | | | | | / | | | | | | | | | | | | | | | | | | | | | | |  | | | | | | | |
| γ-linolenic acid | | 1.022(0.912~1.145) | | | | | | | | 0.904(0.813~1.005) | | | | | | | | | | 0.128 | | | | | | | | | | | | | | | | | | | 1.029(0.914~1.158) | | | | | | | | | 0.903(0.807~1.010) | | | | | | | | | | | | | 0.143 | | | | | | | | | | | | | | | | | | 1.030(0.914~1.160) | | | | | | | | | | 0.918(0.823~1.024) | | | | | | | | | | | | 0.243 | | | | | | | | | | | | | | | | | | | | | |  |  |  |  |  |  |  |  |  |  |  |
| Arachidonic acid | | 1.009(0.994~1.024) | | | | | | | | 0.996(0.984~1.007) | | | | | | | | | | 0.158 | | | | | | | | | | | | | | | | | | | 1.010(0.995~1.025) | | | | | | | | | 0.994(0.983~1.006) | | | | | | | | | | | | | 0.114 | | | | | | | | | | | | | | | | | | 1.010(0.995~1.026) | | | | | | | | | | 0.994(0.983~1.006) | | | | | | | | | | | | 0.098 | | | | | | | | | | | | | | | | | | | | | |  |  |  |  |  |  |  |  |  |  |  |
| Docosatetraenoic acid | | 0.841(0.532~1.332) | | | | | | | | 1.182(0.841~1.662) | | | | | | | | | | 0.234 | | | | | | | | | | | | | | | | | | | 0.783(0.484~1.266) | | | | | | | | | 1.232(0.858~1.770) | | | | | | | | | | | | | 0.164 | | | | | | | | | | | | | | | | | | 0.778(0.482~1.258) | | | | | | | | | | 1.216(0.849~1.743) | | | | | | | | | | | | 0.171 | | | | | | | | | | | | | | | | | | | | | |  |  |  |  |  |  |  |  |  |  |  |
| Eicosadienoic acid | | 1.108(0.880~1.396) | | | | | | | | **0.721(0.542~0.958) *** | | | | | | | | | | | | | | | | | | | | | **0.020*** | | | | | | | | | | | | | 1.097(0.866~1.389) | | | | | | | | | **0.689(0.511~0.928) *** | | | | | | | | | | | | | | | | | | **0.016*** | | | | | | | | | | | | 1.117(0.882~1.415) | | | | | | | | | **0.714(0.532~0.958) *** | | | | | | | | | | | | | | | | | | **0.026*** | | | | | | | | | | | |  |  |  |  |  |  |  |  |  |  |  |  |
| Eicosatrienoic acid | | 0.992(0.969~1.016) | | | | | | | | 1.009(0.985~1.035) | | | | | | | | | | 0.319 | | | | | | | | | | | | | | | | | | | 0.994(0.970~1.018) | | | | | | | | | 1.014(0.987~1.042) | | | | | | | | | | | | | 0.289 | | | | | | | | | | | | | | | | | | 0.993(0.969~1.018) | | | | | | | | | | 1.011(0.985~1.038) | | | | | | | | | | | | 0.366 | | | | | | | | | | | | | | | | | | | | | |  |  |  |  |  |  |  |  |  |  |  |
| Linoleic acid | | 1.002(0.997~1.008) | | | | | | | | 1.005(1.000~1.011) | | | | | | | | | | 0.431 | | | | | | | | | | | | | | | | | | | 1.002(0.997~1.008) | | | | | | | | | 1.006(1.000~1.012) | | | | | | | | | | | | | 0.393 | | | | | | | | | | | | | | | | | | 1.001(0.996~1.007) | | | | | | | | | | 1.006(1.000~1.011) | | | | | | | | | | | | 0.321 | | | | | | | | | | | | | | | | | | | | | |  |  |  |  |  |  |  |  |  |  |  |
| n-6 PUFAs | | 1.103(0.737~ 1.652) | | | | | | | | 0.789(0.562~1.109) | | | | | | | | | | | | | | | | | / | | | | | | | | | 1.160(0.765~1.759) | | | | | | | | | | | | | | | | 0.765 (0.538~1.089) | | | | | | | | | | | | | | | | | | / | | | | | | | | 1.164(0.765~1.772) | | | | | | | | | | | 0.754(0.529~1.075) | | | | | | | | | | | | | | | | | | / | | | | | | | | | | | | | | | | | | |  |  |  |  |  |  |  |  |
| Arachidic acid | | 0.979(0.575~1.667) | | | | | | | | 1.016(0.611~1.692) | | | | | | | | | | 0.907 | | | | | | | | | | | | | | | | | | | 1.034(0.589~1.814) | | | | | | | | | 0.925(0.545~1.569) | | | | | | | | | | | | | 0.933 | | | | | | | | | | | | | | | | | | 1.029(0.585~1.809) | | | | | | | | | | 0.894(0.529~1.511) | | | | | | | | | | | | 0.833 | | | | | | | | | | | | | | | | | | | | | |  |  |  |  |  |  |  |  |  |  |  |
| Docosanoic acid | | 1.042(0.875~1.240) | | | | | | | | 0.950(0.803~1.123) | | | | | | | | | | 0.433 | | | | | | | | | | | | | | | | | | | 1.042(0.875~1.242) | | | | | | | | | 0.943(0.790~1.125) | | | | | | | | | | | | | 0.480 | | | | | | | | | | | | | | | | | | 1.046(0.879~1.246) | | | | | | | | | | 0.946(0.794~1.127) | | | | | | | | | | | | 0.455 | | | | | | | | | | | | | | | | | | | | | |  |  |  |  |  |  |  |  |  |  |  |
| Eicosaenoic acid | | 1.140(0.973~1.336) | | | | | | | | 0.772(0.549~1.086) | | | | | | | | | | 0.046 | | | | | | | | | | | | | | | | | | | 1.129(0.960~1.328) | | | | | | | | | 0.737(0.517~1.051) | | | | | | | | | | | | | 0.039 | | | | | | | | | | | | | | | | | | 1.146(0.972~1.351) | | | | | | | | | | 0.703(0.488~1.012) | | | | | | | | | | | | 0.018 | | | | | | | | | | | | | | | | | | | | | |  |  |  |  |  |  |  |  |  |  |  |
| Hexadecenoic acid | | 1.037(0.975~1.103) | | | | | | | | 1.013(0.957~1.072) | | | | | | | | | | 0.577 | | | | | | | | | | | | | | | | | | | 1.040(0.976~1.108) | | | | | | | | | 1.014(0.954~1.077) | | | | | | | | | | | | | 0.552 | | | | | | | | | | | | | | | | | | 1.035(0.971~1.103) | | | | | | | | | | 1.012(0.953~1.074) | | | | | | | | | | | | 0.565 | | | | | | | | | | | | | | | | | | | | | |  |  |  |  |  |  |  |  |  |  |  |
| Hexadecanoic acid | | 0.999(0.988~1.010) | | | | | | | | 0.995(0.986~1.004) | | | | | | | | | | 0.636 | | | | | | | | | | | | | | | | | | | 0.999(0.987~1.010) | | | | | | | | | 0.994(0.985~1.004) | | | | | | | | | | | | | 0.562 | | | | | | | | | | | | | | | | | | 1.001(0.990~1.012) | | | | | | | | | | 0.994(0.985~1.003) | | | | | | | | | | | | 0.386 | | | | | | | | | | | | | | | | | | | | | |  |  |  |  |  |  |  |  |  |  |  |
| Lignoceric acid | | 0.887(0.657~1.199) | | | | | **1.501(1.132~1.989) **** | | | | | | | | | | | | | | | | | | | | | | **0.012*** | | | | | | | | | | | | 0.891(0.648~1.225) | | | | | | | | **1.515(1.125~2.041) **** | | | | | | | | | | | | | | | | | | | | | | | | **0.018*** | | | | | | | | | | | | 0.902(0.657~1.238) | | | | | | | | | | | **1.483(1.105~1.990) **** | | | | | | | | | | | | | | | | | | **0.029*** | | | |  |  |  |  |  |  |  |  |  |  |  |  |  |  |  |  |
| Octadecanoic acid | | 0.997(0.972~1.022) | | | | | | | | 1.012(0.989~1.034) | | | | | | | | | | 0.367 | | | | | | | | | | | | | | | | | | | 0.997(0.971~1.023) | | | | | | | | | 1.007(0.984~1.030) | | | | | | | | | | | | | 0.573 | | | | | | | | | | | | | | | | | | 0.997(0.971~1.023) | | | | | | | | | | 1.009(0.986~1.032) | | | | | | | | | | | | 0.469 | | | | | | | | | | | | | | | | | | | | | |  |  |  |  |  |  |  |  |  |  |  |
| Octadecenoic acid | | 0.998(0.989~1.007) | | | | | | | | 1.003(0.995~1.012) | | | | | | | | | | 0.352 | | | | | | | | | | | | | | | | | | | 0.998(0.989~1.008) | | | | | | | | | 1.005(0.996~1.014) | | | | | | | | | | | | | 0.291 | | | | | | | | | | | | | | | | | | 0.997(0.988~1.007) | | | | | | | | | | 1.006(0.997~1.015) | | | | | | | | | | | | 0.187 | | | | | | | | | | | | | | | | | | | | | |  |  |  |  |  |  |  |  |  |  |  |
| Tetracosenic acid | | 0.980(0.934~1.028) | | | | | | | | 1.047(0.994~1.103) | | | | | | | | | | 0.071 | | | | | | | | | | | | | | | | | | | 0.981(0.933~1.030) | | | | | | | | | 1.059(1.004~1.117) * | | | | | | | | | | | | | | | | | | 0.054 | | | | | | | | | | | | | | | | | | | 0.978(0.931~1.028) | | | | | | | | | | | **1.061(1.005~1.119) *** | | | | | | | | | | | | | | | | | **0.040*** | | | | | | | | |  |  |  |  |  |  |  |  |  |  |  |  |
| Tetradecanoic acid | | 1.004(0.918~1.097) | | | | | | | | 1.039(0.961~1.123) | | | | | | | | | | 0.581 | | | | | | | | | | | | | | | | | | | 1.005(0.915~1.104) | | | | | | | | | 1.046(0.965~1.133) | | | | | | | | | | | | | 0.602 | | | | | | | | | | | | | | | | | | 0.993(0.902~1.094) | | | | | | | | | | 1.045(0.965~1.131) | | | | | | | | | | | | 0.563 | | | | | | | | | | | | | | | | | | | | | |  |  |  |  |  |  |  |  |  |  |  |
| other fatty acid mixtures | | 0.891(0.546~1.454) | | | | | | | | 0.723(0.488~1.071) | | | | | | | | | | | | | | | | | / | | | | | | | | | 0.911(0.550~1.509) | | | | | | | | | | | | | | | | 0.660(0.435~1.003) | | | | | | | | | | | | | | | | | | / | | | | | | | | 0.882 (0.530~1.470) | | | | | | | | | | | | | | | | | | **0.656(0.432~ -0.998) *** | | | | | | | | | | | | | | | | | | | | / | | | |  |  |  |  |  |  |  |  |  |  |  |  |  |  |
| ∑fatty acid mixtures | 0.853(0.480~1.515) | | | | | | | | | | 0.760(0.479~1.205) | | | | | | | | | | | | | | | | | / | | | | | | 0.861(0.476~1.557) | | | | | | | | | | | | | 0.676(0.411~1.111) | | | | | | | | | | | | | | | | | | / | | | | | | | | | | | | 0.827 (0.452~1.511) | | | | | | | | | | | | | | | | 0.675(0.411~1.111) | | | | | | | | | | | | | | | | / | | | | | | | | | | | | | | | | | | | |  |  |  |  |  |
|  | **high stable increasing vs. stable increasing (n=59 vs. n=207)3 vs.2** | | | | | | | **high stable increasing vs. stable increasing (n=33 vs. n=187)3 vs.2** | | | | | | | | |  | | | | | | | | | | | | | | | | | | **high stable increasing vs. stable increasing (n=59 vs. n=207)3 vs.2** | | | | | | | | | | | **high stable increasing vs. stable increasing (n=33 vs. n=187)3 vs.2** | | | | | | | | | | | |  | | | | | | | | | | | | | | | | | | | **high stable increasing vs. stable increasing (n=59 vs. n=207)3 vs.2** | | | | | | | | | | | **high stable increasing vs. stable increasing (n=33 vs. n=187)3 vs.2** | | | | | | | | | | |  | | | | | | | | | | | | | | | | | | | | | |  |  |  |  |  |  |  |  |  |  |  |  |  |
| Docosahexaenoic acid | 0.992(0.970~1.014) | | | | | | | | 1.017(0.987~1.049) | | | | | | | | | | 0.169 | | | | | | | | | | | | | | | | | | | 0.994(0.972~1.017) | | | | | | | | | 1.013(0.982~1.045) | | | | | | | | | | | | | 0.208 | | | | | | | | | | | | | | | | | | | | | 0.995(0.972~1.017) | | | | | | | | | 1.013(0.983~1.045) | | | | | | | | | | 0.160 | | | | | | | | | | | | | | | | | | | | | | | | | | |  |  |  |  |  |  |  |
| Eicosapentaenoic acid | 1.010(0.960~1.062) | | | | | | | | 0.962(0.874~1.060) | | | | | | | | | | 0.402 | | | | | | | | | | | | | | | | | | | 1.015(0.962~1.071) | | | | | | | | | 0.971(0.875~1.077) | | | | | | | | | | | | | 0.397 | | | | | | | | | | | | | | | | | | | | | 1.015(0.963~1.069) | | | | | | | | | 0.969(0.874~1.075) | | | | | | | | | | 0.367 | | | | | | | | | | | | | | | | | | | | | | | | | | |  |  |  |  |  |  |  |
| α-linolenic acid | 1.016(0.977~1.057) | | | | | | | | 1.001(0.949~1.057) | | | | | | | | | | 0.664 | | | | | | | | | | | | | | | | | | | 1.017(0.976~1.060) | | | | | | | | | 1.003(0.947~1.063) | | | | | | | | | | | | | 0.660 | | | | | | | | | | | | | | | | | | | | | 1.016(0.975~1.058) | | | | | | | | | 1.011(0.956~1.069) | | | | | | | | | | 0.711 | | | | | | | | | | | | | | | | | | | | | | | | | | |  |  |  |  |  |  |  |
| Docosapentenoic acid | 0.856(0.657~1.115) | | | | | | | | 1.260(0.904~1.757) | | | | | | | | | | 0.076 | | | | | | | | | | | | | | | | | | | 0.828(0.631~1.088) | | | | | | | | | 1.217(0.849~1.744) | | | | | | | | | | | | | 0.074 | | | | | | | | | | | | | | | | | | | | | 0.871(0.666~1.139) | | | | | | | | | 1.230(0.862~1.756) | | | | | | | | | | 0.104 | | | | | | | | | | | | | | | | | | | | | | | | | | |  |  |  |  |  |  |  |
| n-3 PUFAs | | | 0.995(0.685~1.447) | | | | | | | | | | 1.192(0.750~1.897) | | | | | | | | | | | | | / | | | | | | | | | | | | 0.962 (0.652~1.420) | | | | | | | | | | | | | | | | 1.397 (0.842~2.318) | | | | | | | | | | | | | | | / | | | | | | | | | | | 0.943(0.637~1.397) | | | | | | | | | | | | | | | | 1.359(0.814~2.267) | | | | | | | | | | | | | | | / | | | | | | | | | | | | | | | | | | | | | | |
| γ-linolenic acid | | | 0.956(0.853~1.072) | | | | | | | | | | 0.906(0.776~1.059) | | | | | | | | | | | 0.597 | | | | | | | | | | | | | | | | | | | 0.958(0.852~1.078) | | | | | | | | 0.891(0.756~1.048) | | | | | | | | | | | | 0.548 | | | | | | | | | | | | | | | | | | | | | 0.943(0.834~1.065) | | | | | | | | | 0.893(0.759~1.051) | | | | | | | | | | | 0.789 | | | | | | | | | | | | | | | | | | | | |  |  |  |  |  |  |  |  |  |
| Arachidonic acid | | | 0.998(0.985~1.010) | | | | | | | | | | 0.990(0.974~1.006) | | | | | | | | | | | 0.439 | | | | | | | | | | | | | | | | | | | 0.996(0.983~1.009) | | | | | | | | 0.989(0.973~1.006) | | | | | | | | | | | | 0.604 | | | | | | | | | | | | | | | | | | | | | 0.996(0.983~1.009) | | | | | | | | | 0.988(0.971~1.005) | | | | | | | | | | | 0.503 | | | | | | | | | | | | | | | | | | | | |  |  |  |  |  |  |  |  |  |
| Docosatetraenoic acid | | | 1.136(0.770~1.677) | | | | | | | | | | 1.037(0.658~1.634) | | | | | | | | | | | 0.766 | | | | | | | | | | | | | | | | | | | 1.208(0.810~1.802) | | | | | | | | 1.066(0.659~1.726) | | | | | | | | | | | | 0.726 | | | | | | | | | | | | | | | | | | | | | 1.243(0.829~1.864) | | | | | | | | | 1.131(0.696~1.837) | | | | | | | | | | | 0.737 | | | | | | | | | | | | | | | | | | | | |  |  |  |  |  |  |  |  |  |
| Eicosadienoic acid | | | 0.916(0.729~1.151) | | | | | | | | | | 0.914(0.665~1.257) | | | | | | | | | | | 0.995 | | | | | | | | | | | | | | | | | | | 0.939(0.738~1.194) | | | | | | | | 0.885(0.633~1.236) | | | | | | | | | | | | 0.980 | | | | | | | | | | | | | | | | | | | | | 0.922(0.727~1.170) | | | | | | | | | 0.879(0.629~1.228) | | | | | | | | | | | 0.803 | | | | | | | | | | | | | | | | | | | | |  |  |  |  |  |  |  |  |  |
| Eicosatrienoic acid | | | 0.996(0.974~1.019) | | | | | | | | | | 1.002(0.969~1.036) | | | | | | | | | | | 0.781 | | | | | | | | | | | | | | | | | | | 0.993(0.969~1.018) | | | | | | | | 1.008(0.972~1.045) | | | | | | | | | | | | 0.955 | | | | | | | | | | | | | | | | | | | | | 0.996(0.972~1.020) | | | | | | | | | 1.008(0.972~1.045) | | | | | | | | | | | 0.815 | | | | | | | | | | | | | | | | | | | | |  |  |  |  |  |  |  |  |  |
| Linoleic acid | | | 1.002(0.996~1.007) | | | | | | | | | | 1.004(0.997~1.011) | | | | | | | | | | | 0.558 | | | | | | | | | | | | | | | | | | | 1.002(0.997~1.007) | | | | | | | | 1.005(0.997~1.012) | | | | | | | | | | | | 0.622 | | | | | | | | | | | | | | | | | | | | | 1.002(0.997~1.008) | | | | | | | | | 1.005(0.997~1.012) | | | | | | | | | | | 0.852 | | | | | | | | | | | | | | | | | | | | |  |  |  |  |  |  |  |  |  |
| n-6 PUFAs | | | 1.096(0.763~1.574) | | | | | | | | | | 0.929(0.583~1.481) | | | | | | | | | | | | | | | | | / | | | | | | | | 1.051 (0.726~1.521) | | | | | | | | | | | | | 1.044(0.626~1.740) | | | | | | | | | | | | | | / | | | | | | | | | | | | | | | 1.041 (0.718~1.510) | | | | | | | | | | | | | 0.965(0.573~1.625) | | | | | | | | | | | | | | | | | | / | | | | | | | | | | | | | | | | |  |  |  |  |  |  |
| Arachidic acid | | | 0.868(0.534~1.410) | | | | | | | | | | 0.628(0.336~1.174) | | | | | | | | | | | 0.424 | | | | | | | | | | | | | | | | | | | 0.826(0.498~1.369) | | | | | | | | 0.599(0.306~1.171) | | | | | | | | | | | | 0.424 | | | | | | | | | | | | | | | | | | | | | 0.807(0.487~1.338) | | | | | | | | | 0.602(0.308~1.177) | | | | | | | | | | | 0.583 | | | | | | | | | | | | | | | | | | | | |  |  |  |  |  |  |  |  |  |
| Docosanoic acid | | | 0.974(0.841~1.128) | | | | | | | | | | 1.134(0.922~1.395) | | | | | | | | | | | 0.247 | | | | | | | | | | | | | | | | | | | 0.965(0.826~1.126) | | | | | | | | 1.110(0.896~1.374) | | | | | | | | | | | | 0.238 | | | | | | | | | | | | | | | | | | | | | 0.972(0.836~1.130) | | | | | | | | | 1.100(0.890~1.361) | | | | | | | | | | | 0.329 | | | | | | | | | | | | | | | | | | | | |  |  |  |  |  |  |  |  |  |
| Eicosaenoic acid | | | 1.061(0.888~1.268) | | | | | | | | | | 1.051(0.760~1.453) | | | | | | | | | | | 0.978 | | | | | | | | | | | | | | | | | | | 1.102(0.916~1.324) | | | | | | | | 1.038(0.725~1.487) | | | | | | | | | | | | 0.913 | | | | | | | | | | | | | | | | | | | | | 1.122(0.941~1.339) | | | | | | | | | 1.039(0.730~1.477) | | | | | | | | | | | 0.833 | | | | | | | | | | | | | | | | | | | | |  |  |  |  |  |  |  |  |  |
| Hexadecenoic acid | | | 0.998(0.939~1.061) | | | | | | | | | | 1.044(0.969~1.124) | | | | | | | | | | | 0.357 | | | | | | | | | | | | | | | | | | | 1.002(0.938~1.069) | | | | | | | | 1.038(0.959~1.122) | | | | | | | | | | | | 0.420 | | | | | | | | | | | | | | | | | | | | | 1.009(0.946~1.077) | | | | | | | | | 1.039(0.960~1.124) | | | | | | | | | | | 0.557 | | | | | | | | | | | | | | | | | | | | |  |  |  |  |  |  |  |  |  |
| Hexadecanoic acid | | | 0.999(0.989~1.009) | | | | | | | | | | 0.995(0.983~1.006) | | | | | | | | | | | 0.579 | | | | | | | | | | | | | | | | | | | 0.998(0.988~1.009) | | | | | | | | 0.994(0.982~1.007) | | | | | | | | | | | | 0.639 | | | | | | | | | | | | | | | | | | | | | 0.998(0.988~1.008) | | | | | | | | | 0.994(0.982~1.007) | | | | | | | | | | | 0.759 | | | | | | | | | | | | | | | | | | | | |  |  |  |  |  |  |  |  |  |
| Lignoceric acid | | | 1.215(0.930~1.589) | | | | | | | | | | 0.809(0.572~1.144) | | | | | | | | | | | 0.068 | | | | | | | | | | | | | | | | | | | 1.239(0.938~1.635) | | | | | | | | 0.867(0.602~1.250) | | | | | | | | | | | | 0.066 | | | | | | | | | | | | | | | | | | | | | 1.189(0.903~1.564) | | | | | | | | | 0.875(0.611~1.253) | | | | | | | | | | | 0.103 | | | | | | | | | | | | | | | | | | | | |  |  |  |  |  |  |  |  |  |
| Octadecanoic acid | | | 1.021(0.999~1.043) | | | | | | | | | | 1.014(0.986~1.043) | | | | | | | | | | | 0.676 | | | | | | | | | | | | | | | | | | | 1.027(1.003~1.051) | | | | | | | | 1.014(0.985~1.044) | | | | | | | | | | | | 0.697 | | | | | | | | | | | | | | | | | | | | | 1.025(1.001~1.050) | | | | | | | | | 1.012(0.983~1.042) | | | | | | | | | | | 0.695 | | | | | | | | | | | | | | | | | | | | |  |  |  |  |  |  |  |  |  |
| Octadecenoic acid | | | 0.997(0.989~1.005) | | | | | | | | | | 0.997(0.987~1.008) | | | | | | | | | | | 0.984 | | | | | | | | | | | | | | | | | | | 0.995(0.986~1.004) | | | | | | | | 0.998(0.987~1.009) | | | | | | | | | | | | 0.842 | | | | | | | | | | | | | | | | | | | | | 0.995(0.986~1.003) | | | | | | | | | 0.998(0.987~1.010) | | | | | | | | | | | 0.685 | | | | | | | | | | | | | | | | | | | | |  |  |  |  |  |  |  |  |  |
| Tetracosenic acid | | | 1.009(0.969~1.049) | | | | | | | | | | 1.006(0.943~1.072) | | | | | | | | | | | 0.928 | | | | | | | | | | | | | | | | | | | 1.009(0.968~1.051) | | | | | | | | 1.016(0.949~1.088) | | | | | | | | | | | | 0.976 | | | | | | | | | | | | | | | | | | | | | 1.005(0.965~1.047) | | | | | | | | | 1.019(0.952~1.090) | | | | | | | | | | | 0.973 | | | | | | | | | | | | | | | | | | | | |  |  |  |  |  |  |  |  |  |
| Tetradecanoic acid | | | 0.983(0.902~1.072) | | | | | | | | | | 0.977(0.868~1.100) | | | | | | | | | | | 0.906 | | | | | | | | | | | | | | | | | | | 0.971(0.882~1.068) | | | | | | | | 0.982(0.866~1.114) | | | | | | | | | | | | 0.957 | | | | | | | | | | | | | | | | | | | | | 0.967(0.879~1.064) | | | | | | | | | 0.964(0.849~1.096) | | | | | | | | | | | 0.994 | | | | | | | | | | | | | | | | | | | | |  |  |  |  |  |  |  |  |  |
| other fatty acid mixtures | | | 0.924 (0.606~1.410) | | | | | | | | | | 1.172(0.699~1.966) | | | | | | | | | | | | | / | | | | | | | | | | | | 0.855 (0.553~1.323) | | | | | | | | | | | | | | | | 1.383(0.792~2.413) | | | | | | | | | | | | | | | / | | | | | | | | | | | 0.843(0.543~1.307) | | | | | | | | | | | | | | | | 1.352(0.773~2.367) | | | | | | | | | | | | | | | / | | | | | | | | | | | | | | | | | | | | | | |
| ∑fatty acid mixtures | | | 0.900(0.552~1.469) | | | | | | | | | | 1.260(0.686~2.312) | | | | | | | | | | | | | / | | | | | | | | | | | | 0.818(0.492~1.361) | | | | | | | | | | | | | | | | 1.688(0.854~3.339) | | | | | | | | | | | | | | | / | | | | | | | | | | | | | | | 0.810(0.486~1.350) | | | | | | | | | | | | 1.621 (0.809~3.248) | | | | | | | | | | | | | | | / | | | | | | | | | | | | | | | | | | | | | | |
|  | | | **dramatically falling vs. stable increasing (n=29 vs. n=207) 4 vs.2** | | | | | | | | | | **dramatically falling vs. stable increasing (n=24 vs. n=187) 4 vs.2** | | | | | | | | | | |  | | | | | | | | | | | | | | | | | | | **dramatically falling vs. stable increasing (n=29 vs. n=207) 4 vs.2** | | | | | | | | **dramatically falling vs. stable increasing (n=24 vs. n=187) 4 vs.2** | | | | | | | | | | | |  | | | | | | | | | | | | | | | | | | | | | **dramatically falling vs. stable increasing (n=29 vs. n=207) 4 vs.2** | | | | | | | | | **dramatically falling vs. stable increasing (n=24 vs. n=187) 4 vs.2** | | | | | | | | | | |  | | | | | | | | | | | | | | | | | | | | |  |  |  |  |  |  |  |  |  |
| Docosahexaenoic acid | | | | 1.006(0.973~1.040) | | | | | | | | | | | 1.007(0.970~1.045) | | | | | | | | | 0.968 | | | | | | | | | | | | | | | | | | | 0.996(0.960~1.033) | | | | | | | | 1.004(0.966~1.044) | | | | | | | | | | | | 0.767 | | | | | | | | | | | | | | | | | | | | | 1.000(0.965~1.037) | | | | | | | | | 1.004(0.966~1.044) | | | | | | | | | | | 0.811 | | | | | | | | | | | | | | | | | | | | |  |  |  |  |  |  |  |  |  |
| Eicosapentaenoic acid | | | 0.923(0.825~1.034) | | | | | | | | | | 0.977(0.863~1.107) | | | | | | | | | | | 0.453 | | | | | | | | | | | | | | | | | | | 0.926(0.820~1.046) | | | | | | | | 0.976(0.855~1.114) | | | | | | | | | | | | 0.477 | | | | | | | | | | | | | | | | | | | | | 0.928(0.822~1.048) | | | | | | | | | 0.977(0.858~1.113) | | | | | | | | | | | 0.412 | | | | | | | | | | | | | | | | | | | | |  |  |  |  |  |  |  |  |  |
| α-linolenic acid | | | 1.034(0.973~1.100) | | | | | | | | | | 1.044(0.977~1.115) | | | | | | | | | | | 0.884 | | | | | | | | | | | | | | | | | | | 1.051(0.985~1.120) | | | | | | | | 1.041(0.970~1.117) | | | | | | | | | | | | 0.948 | | | | | | | | | | | | | | | | | | | | | 1.048(0.983~1.117) | | | | | | | | | 1.039(0.967~1.115) | | | | | | | | | | | 0.885 | | | | | | | | | | | | | | | | | | | | |  |  |  |  |  |  |  |  |  |
| Docosapentenoic acid | | | 0.694(0.483~0.996) | | | | | | | | | | 1.049(0.702~1.567) | | | | | | | | | | | 0.160 | | | | | | | | | | | | | | | | | | | 0.614(0.400~0.943) | | | | | | | | 1.045(0.680~1.606) | | | | | | | | | | | | 0.078 | | | | | | | | | | | | | | | | | | | | | 0.674(0.447~1.018) | | | | | | | | | 1.040(0.678~1.596) | | | | | | | | | | | 0.094 | | | | | | | | | | | | | | | | | | | | |  |  |  |  |  |  |  |  |  |
| n-3 PUFAs | | | 1.153 (0.682~1.947) | | | | | | | | | | 0.954 (0.553~1.646) | | | | | | | | | | | | | | | | | / | | | | | | | | 1.141(0.678~1.920) | | | | | | | | | | | | | | | | 0.941(0.537~1.651) | | | | | | | | | | | | | | | | | | / | | | | | | | | 1.181(0.698~1.998) | | | | | | | | | | | | | | | | 0.937(0.533~1.648) | | | | | | | | | | | | | | | | / | | | | | | | | |  | | | | | | | | | | | | |
| γ-linolenic acid | | | 0.880(0.720~1.074) | | | | | | | | | | 0.880(0.721~1.076) | | | | | | | | | | | 0.985 | | | | | | | | | | | | | | | | | | | 0.857(0.682~1.078) | | | | | | | | 0.868(0.706~1.068) | | | | | | | | | | | | 0.982 | | | | | | | | | | | | | | | | | | | | | 0.854(0.679~1.074) | | | | | | | | | 0.869(0.707~1.067) | | | | | | | | | | | 0.996 | | | | | | | | | | | | | | | | | | | | |  |  |  |  |  |  |  |  |  |
| Arachidonic acid | | | 1.012(0.995~1.029) | | | | | | | | | | 1.000(0.981~1.019) | | | | | | | | | | | 0.393 | | | | | | | | | | | | | | | | | | | 1.011(0.993~1.029) | | | | | | | | 1.001(0.982~1.021) | | | | | | | | | | | | 0.414 | | | | | | | | | | | | | | | | | | | | | 1.010(0.992~1.028) | | | | | | | | | 1.002(0.982~1.021) | | | | | | | | | | | 0.408 | | | | | | | | | | | | | | | | | | | | |  |  |  |  |  |  |  |  |  |
| Docosatetraenoic acid | | | 0.716(0.431~1.189) | | | | | | | | | | 0.951(0.552~1.636) | | | | | | | | | | | 0.405 | | | | | | | | | | | | | | | | | | | 0.649(0.367~1.147) | | | | | | | | 0.939(0.541~1.631) | | | | | | | | | | | | 0.283 | | | | | | | | | | | | | | | | | | | | | 0.667(0.381~1.169) | | | | | | | | | 0.926(0.531~1.616) | | | | | | | | | | | 0.326 | | | | | | | | | | | | | | | | | | | | |  |  |  |  |  |  |  |  |  |
| Eicosadienoic acid | | | 1.038(0.766~1.406) | | | | | | | | | | 1.062(0.707~1.595) | | | | | | | | | | | 0.967 | | | | | | | | | | | | | | | | | | | 1.057(0.745~1.499) | | | | | | | | 1.033(0.680~1.567) | | | | | | | | | | | | 0.928 | | | | | | | | | | | | | | | | | | | | | 1.063(0.752~1.502) | | | | | | | | | 1.032(0.681~1.565) | | | | | | | | | | | 0.815 | | | | | | | | | | | | | | | | | | | | |  |  |  |  |  |  |  |  |  |
| Eicosatrienoic acid | | | 0.984(0.953~1.015) | | | | | | | | | | 1.015(0.974~1.058) | | | | | | | | | | | 0.291 | | | | | | | | | | | | | | | | | | | 0.987(0.953~1.022) | | | | | | | | 1.023(0.979~1.069) | | | | | | | | | | | | 0.220 | | | | | | | | | | | | | | | | | | | | | 0.989(0.955~1.024) | | | | | | | | | 1.022(0.979~1.068) | | | | | | | | | | | 0.180 | | | | | | | | | | | | | | | | | | | | |  |  |  |  |  |  |  |  |  |
| Linoleic acid | | | 0.998(0.991~1.005) | | | | | | | | | | 1.003(0.994~1.011) | | | | | | | | | | | 0.314 | | | | | | | | | | | | | | | | | | | 0.998(0.990~1.005) | | | | | | | | 1.003(0.994~1.012) | | | | | | | | | | | | 0.305 | | | | | | | | | | | | | | | | | | | | | 0.999(0.992~1.007) | | | | | | | | | 1.003(0.994~1.012) | | | | | | | | | | | 0.378 | | | | | | | | | | | | | | | | | | | | |  |  |  |  |  |  |  |  |  |
| n-6 PUFAs | | | 0.853 (0.517~1.407) | | | | | | | | | | 0.709 (0.417~1.206) | | | | | | | | | | | / | | | | | | | | | | | | | | 0.851(0.513~1.412) | | | | | | | | | | | | | | | | 0.712(0.411~1.233) | | | | | | | | | | | | | | | | | | / | | | | | | | | | 0.869(0.521~1.448) | | | | | | | | | | | | | | | 0.689(0.397~1.195) | | | | | | | | | | | | | | | | / | | | | | | | | |  | | | | | | | | | | | |  |
| Arachidic acid | | | **2.459(1.229~4.922) *** | | | | | | | | | | | | | 0.846(0.400~1.790) | | | | | | | | | | **0.044*** | | | | | | | | | | | | | | | | | | | **3.211(1.434~7.190) **** | | | | | | | | | | | | 0.808(0.376~1.734) | | | | | | | | | | | | | | | | | **0.026*** | | | | | | | | | | | | | **2.684(1.221~5.900) *** | | | | | | | | | | | 0.812(0.380~1.734) | | | | | | | | | | | | | | | | | **0.043*** | | | |  |  |  |  |  |  |  |  |  |  |  |  |  |  |  |
| Docosanoic acid | | | | 0.910(0.701~1.181) | | | | | | | | | | | 1.007(0.768~1.320) | | | | | | | | 0.693 | | | | | | | | | | | | | | | | | | | | 0.868(0.648~1.161) | | | | | | | | 0.988(0.749~1.303) | | | | | | | | | | | | 0.655 | | | | | | | | | | | | | | | | | | | | | 0.898(0.673~1.198) | | | | | | | | | 0.987(0.750~1.300) | | | | | | | | | | | 0.768 | | | | | | | | | | | | | | | | | | | | | | | | | |  |  |  |  |
| Eicosaenoic acid | | | | 0.896(0.576~1.394) | | | | | | | | | | | 0.634(0.328~1.224) | | | | | | | | 0.399 | | | | | | | | | | | | | | | | | | | | 0.808(0.496~1.317) | | | | | | | | 0.629(0.314~1.259) | | | | | | | | | | | | 0.508 | | | | | | | | | | | | | | | | | | | | | 0.850(0.534~1.352) | | | | | | | | | 0.633(0.318~1.262) | | | | | | | | | | | 0.549 | | | | | | | | | | | | | | | | | | | | | | | | | |  |  |  |  |
| Hexadecenoic acid | | | | 0.972(0.886~1.067) | | | | | | | | | | | 1.017(0.920~1.125) | | | | | | | | 0.531 | | | | | | | | | | | | | | | | | | | | 0.952(0.861~1.053) | | | | | | | | 1.012(0.912~1.123) | | | | | | | | | | | | 0.382 | | | | | | | | | | | | | | | | | | | | | 0.979(0.890~1.076) | | | | | | | | | 1.016(0.917~1.125) | | | | | | | | | | | 0.304 | | | | | | | | | | | | | | | | | | | | | | | | | |  |  |  |  |
| Hexadecanoic acid | | | | 1.010(0.995~1.025) | | | | | | | | | | | 0.990(0.975~1.005) | | | | | | | | 0.067 | | | | | | | | | | | | | | | | | | | | 1.013(0.997~1.029) | | | | | | | | 0.990(0.974~1.006) | | | | | | | | | | | | 0.033 | | | | | | | | | | | | | | | | | | | | | 1.009(0.994~1.025) | | | | | | | | | 0.990(0.974~1.006) | | | | | | | | | | | 0.077 | | | | | | | | | | | | | | | | | | | | | | | | | |  |  |  |  |
| Lignoceric acid | | | | 1.471(0.985~2.196) | | | | | | | | | | | 0.948(0.626~1.435) | | | | | | | | 0.167 | | | | | | | | | | | | | | | | | | | | 1.636(1.025~2.611) | | | | | | | | 0.984(0.634~1.528) | | | | | | | | | | | | 0.098 | | | | | | | | | | | | | | | | | | | | | 1.480(0.950~2.305) | | | | | | | | | 0.988(0.639~1.529) | | | | | | | | | | | 0.124 | | | | | | | | | | | | | | | | | | | | | | | | | |  |  |  |  |
| Octadecanoic acid | | | | 0.994(0.961~1.029) | | | | | | | | | | | 1.014(0.979~1.049) | | | | | | | | 0.272 | | | | | | | | | | | | | | | | | | | | 0.998(0.962~1.036) | | | | | | | | 1.011(0.976~1.047) | | | | | | | | | | | | 0.570 | | | | | | | | | | | | | | | | | | | | | 0.999(0.963~1.036) | | | | | | | | | 1.011(0.976~1.047) | | | | | | | | | | | 0.671 | | | | | | | | | | | | | | | | | | | | | | | | | |  |  |  |  |
| Octadecenoic acid | | | | 0.992(0.979~1.005) | | | | | | | | | | | 1.001(0.987~1.015) | | | | | | | | 0.268 | | | | | | | | | | | | | | | | | | | | 0.990(0.975~1.004) | | | | | | | | 1.002(0.987~1.017) | | | | | | | | | | | | 0.280 | | | | | | | | | | | | | | | | | | | | | 0.987(0.973~1.002) | | | | | | | | | 1.002(0.987~1.016) | | | | | | | | | | | 0.267 | | | | | | | | | | | | | | | | | | | | | | | | | |  |  |  |  |
| Tetracosenic acid | | | | 0.972(0.907~1.042) | | | | | | | | | | | 0.982(0.901~1.071) | | | | | | | | 0.836 | | | | | | | | | | | | | | | | | | | | 0.976(0.904~1.054) | | | | | | | | 0.986(0.903~1.076) | | | | | | | | | | | | 0.985 | | | | | | | | | | | | | | | | | | | | | 0.983(0.911~1.060) | | | | | | | | | 0.985(0.902~1.075) | | | | | | | | | | | 0.873 | | | | | | | | | | | | | | | | | | | | | | | | | |  |  |  |  |
| Tetradecanoic acid | | | | 1.095(0.996~1.204) | | | | | | | | | | | 1.000(0.864~1.156) | | | | | | | | 0.303 | | | | | | | | | | | | | | | | | | | | 1.107(0.996~1.229) | | | | | | | | 1.007(0.868~1.168) | | | | | | | | | | | | 0.271 | | | | | | | | | | | | | | | | | | | | | 1.107(0.995~1.232) | | | | | | | | | 1.005(0.866~1.166) | | | | | | | | | | | 0.144 | | | | | | | | | | | | | | | | | | | | | | | | | |  |  |  |  |
| other fatty acid  mixtures | | | | | | 1.318 (0.724~2.399) | | | | | | 0.829(0.447~1.536) | | | | | | | | | | | | | / | | | | | | | | 1.311 (0.709~2.423) | | | | | | | | | | | | | | | | | | | | | | 0.860 (0.454~1.630) | | | | | | | | | | | | / | | | | | | | | 1.315(0.707~2.448) | | | | | | | | | | | | | | | | | | | | 0.866(0.458~1.638) | | | | | | | | | | | / | | | | | | | | | | | | | | | | | | | | | | | | |  | | |
| ∑fatty acid mixtures | | | | | | 0.971(0.480~1.963) | | | | | | 0.858(0.418~1.762) | | | | | | | | | | | | | / | | | | | | | 0.901(0.439~1.850) | | | | | | | | | | | | | | | | | | | | | | | | 0.895(0.416 ~1.924) | | | | | | | | | | | | / | | | | | | | | 0.937(0.453~1.940) | | | | | | | | | | | | | | | | | | 0.896(0.415~1.935) | | | | | | | | | | | / | | | | | | | | | | | | | | | | | | | | | | | | | | |  |  |

Note**:** Values are expressed as odds ratios (95% confidence intervals); adjustment models included covariates such as socio-economic status, maternal age, BMI, fetal sex, smoking/drinking and han ethnic groups; IPTW-adjusted models used weighted inverse probabilities based on propensity scores to balance for potential confounders; and *p* indicates gender interaction, **p* < 0.05, ***p* < 0.01, statistically significant difference.

**eTable 7.** Weights for each fatty acids in quantile g-computation (Qgcomp) model in CLIMB study (stratified by sex).

| Fatty acids | Unadjusted | | | | | | Adjusted | | | | IPTW-adjusted | | | | | |  |  |
| --- | --- | --- | --- | --- | --- | --- | --- | --- | --- | --- | --- | --- | --- | --- | --- | --- | --- | --- |
|  | Male | | Female | | | | Male | | | Female | | Male | | | Female | | |  |
|  | **stable falling vs. stable increasing (n=45 vs. n=207) 1 vs.2** | | **stable falling vs. stable increasing (n=71 vs. n=187)**  **1 vs.2** | | | | **stable falling vs. stable increasing (n=45vs. n=207)**  **1 vs.2** | | | **stable falling vs. stable increasing (n=71 vs. n=187)**  **1 vs.2** | | **stable falling vs. stable increasing (n=45 vs. n=207) 1 vs.2** | | | **stable falling vs. stable increasing (n=71vs. n=187)1 vs.2** | | |  |
| Docosahexaenoic acid | 0.097 | | 0.007 | | 0.083 | | | 0.016 | | | 0.111 | | | 0.009 | | |  |  |
| Eicosapentaenoic acid | -0.090 | | 0.073 | | -0.079 | | | 0.036 | | | -0.081 | | | 0.043 | | |  |  |
| α-linolenic acid | 0.113 | | 0.098 | | 0.087 | | | 0.082 | | | 0.096 | | | 0.094 | | |  |  |
| Docosapentenoic acid | 0.127 | | -0.133 | | 0.122 | | | -0.089 | | | 0.108 | | | -0.104 | | |  |  |
| γ-linolenic acid | 0.018 | | -0.130 | | 0.008 | | | -0.110 | | | -0.002 | | | -0.111 | | |  |  |
| Arachidonic acid | -0.023 | | 0.016 | | -0.037 | | | 0.004 | | | -0.017 | | | 0.002 | | |  |  |
| Docosatetraenoic acid | -0.015 | | 0.004 | | -0.022 | | | 0.001 | | | -0.019 | | | -0.007 | | |  |  |
| Eicosadienoic acid | 0.040 | | -0.044 | | 0.007 | | | -0.060 | | | 0.003 | | | -0.050 | | |  |  |
| Eicosatrienoic acid | 0.030 | | 0.014 | | 0.081 | | | 0.038 | | | 0.115 | | | 0.025 | | |  |  |
| Linoleic acid | -0.062 | | 0.109 | | -0.005 | | | 0.144 | | | -0.008 | | | 0.143 | | |  |  |
| Arachidic acid | 0.113 | | -0.144 | | 0.159 | | | -0.146 | | | 0.150 | | | -0.139 | | |  |  |
| Docosanoic acid | -0.138 | | -0.084 | | -0.134 | | | -0.081 | | | -0.123 | | | -0.083 | | |  |  |
| Eicosaenoic acid | -0.063 | | -0.369 | | -0.063 | | | -0.385 | | | -0.061 | | | -0.390 | | |  |  |
| Hexadecenoic acid | 0.168 | | -0.020 | | 0.179 | | | -0.010 | | | 0.165 | | | -0.002 | | |  |  |
| Hexadecanoic acid | 0.154 | | -0.075 | | 0.137 | | | -0.119 | | | 0.128 | | | -0.114 | | |  |  |
| Lignoceric acid | -0.324 | | 0.116 | | -0.359 | | | 0.067 | | | -0.348 | | | 0.085 | | |  |  |
| Octadecanoic acid | 0.141 | | 0.125 | | 0.137 | | | 0.111 | | | 0.124 | | | 0.104 | | |  |  |
| Octadecenoic acid | -0.168 | | 0.115 | | -0.175 | | | 0.148 | | | -0.172 | | | 0.142 | | |  |  |
| Tetracosenic acid | -0.054 | | 0.227 | | -0.063 | | | 0.236 | | | -0.077 | | | 0.240 | | |  |  |
| Tetradecanoic acid | -0.063 | | 0.095 | | -0.064 | | | 0.118 | | | -0.091 | | | 0.114 | | |  |  |
|  | **high stable increasing vs. stable increasing (n=59 vs. n=207)3 vs.2** | | **high stable increasing vs. stable increasing (n=33 vs. n=187)3 vs.2** | | **high stable increasing vs. stable increasing (n=59 vs. n=207)3 vs.2** | | | | | **high stable increasing vs. stable increasing (n=33vs. n=187)3 vs.2** | | | **high stable increasing vs. stable increasing (n=59 vs. n=207)3 vs.2** | | | **high stable increasing vs. stable increasing (n=33 vs. n=187)3 vs.2** | | |
| Docosahexaenoic acid | | -0.085 | | 0.022 | | -0.044 | | | 0.014 | | | -0.049 | | | -0.002 | | |  |
| Eicosapentaenoic acid | -0.120 | | 0.127 | | -0.126 | | | 0.132 | | | -0.124 | | | 0.139 | | |  |  |
| α-linolenic acid | 0.061 | | 0.062 | | 0.091 | | | 0.110 | | | 0.082 | | | 0.127 | | |  |  |
| Docosapentenoic acid | -0.070 | | 0.019 | | -0.032 | | | 0.000 | | | -0.032 | | | -0.017 | | |  |  |
| γ-linolenic acid | 0.013 | | -0.191 | | -0.003 | | | -0.161 | | | 0.012 | | | -0.160 | | |  |  |
| Arachidonic acid | 0.045 | | -0.149 | | 0.035 | | | -0.189 | | | 0.025 | | | -0.200 | | |  |  |
| Docosatetraenoic acid | 0.028 | | 0.073 | | 0.042 | | | 0.118 | | | 0.035 | | | 0.106 | | |  |  |
| Linoleic acid | 0.081 | | 0.234 | | 0.055 | | | 0.247 | | | 0.079 | | | 0.254 | | |  |  |
| Arachidic acid | -0.112 | | -0.136 | | -0.176 | | | -0.088 | | | -0.171 | | | -0.047 | | |  |  |
| Docosanoic acid | -0.008 | | 0.016 | | -0.019 | | | 0.022 | | | -0.022 | | | 0.026 | | |  |  |
| Eicosadienoic acid | 0.016 | | -0.094 | | 0.092 | | | -0.178 | | | 0.082 | | | -0.167 | | |  |  |
| Eicosatrienoic acid | -0.143 | | 0.070 | | -0.141 | | | 0.082 | | | -0.148 | | | 0.077 | | |  |  |
| Eicosaenoic acid | -0.199 | | -0.176 | | -0.170 | | | -0.098 | | | -0.166 | | | -0.106 | | |  |  |
| Hexadecenoic acid | -0.098 | | -0.046 | | -0.060 | | | -0.030 | | | -0.052 | | | -0.001 | | |  |  |
| Hexadecanoic acid | -0.165 | | -0.205 | | -0.164 | | | -0.216 | | | -0.184 | | | -0.224 | | |  |  |
| Lignoceric acid | 0.014 | | 0.025 | | -0.029 | | | 0.083 | | | -0.036 | | | 0.094 | | |  |  |
| Octadecanoic acid | 0.435 | | -0.002 | | 0.511 | | | -0.041 | | | 0.509 | | | -0.075 | | |  |  |
| Octadecenoic acid | 0.097 | | 0.058 | | 0.025 | | | 0.038 | | | 0.028 | | | 0.025 | | |  |  |
| Tetracosenic acid | 0.156 | | 0.157 | | 0.150 | | | 0.110 | | | 0.148 | | | 0.107 | | |  |  |
| Tetradecanoic acid | 0.053 | | 0.138 | | -0.037 | | | 0.045 | | | -0.017 | | | 0.046 | | |  |  |
|  | **dramatically falling vs. stable increasing**  **(n=29 vs. n=207)**  **4 vs.2** | | **dramatically falling vs. stable increasing**  **(n=24 vs. n=187) 4 vs.2** | | **dramatically falling vs. stable increasing**  **(n=29 vs. n=207) 4 vs.2** | | | | | **dramatically falling vs. stable increasing**  **(n=24 vs. n=187)**  **4 vs.2** | | | **dramatically falling vs. stable increasing**  **(n=29 vs. n=207) 4 vs.2** | | | **dramatically falling vs. stable increasing**  **(n=24 vs. n=187) 4 vs.2** | | |
| Docosahexaenoic acid | | -0.011 | | 0.120 | | -0.024 | | | 0.140 | | | -0.020 | | | 0.122 | | |  |
| Eicosapentaenoic acid | -0.103 | | -0.034 | | -0.119 | | | -0.027 | | | -0.108 | | | -0.015 | | |  |  |
| α-linolenic acid | 0.117 | | 0.245 | | 0.112 | | | 0.239 | | | 0.121 | | | 0.254 | | |  |  |
| Docosapentenoic acid | -0.055 | | -0.061 | | -0.046 | | | -0.056 | | | -0.053 | | | -0.079 | | |  |  |
| γ-linolenic acid | -0.123 | | -0.075 | | -0.129 | | | -0.086 | | | -0.131 | | | -0.084 | | |  |  |
| Arachidonic acid | 0.055 | | -0.052 | | 0.079 | | | -0.065 | | | 0.108 | | | -0.063 | | |  |  |
| Docosatetraenoic acid | -0.124 | | -0.031 | | -0.136 | | | -0.027 | | | -0.143 | | | -0.048 | | |  |  |
| Eicosadienoic acid | -0.009 | | 0.003 | | -0.023 | | | -0.008 | | | -0.011 | | | 0.023 | | |  |  |
| Eicosatrienoic acid | -0.003 | | -0.052 | | 0.020 | | | -0.015 | | | 0.014 | | | -0.024 | | |  |  |
| Linoleic acid | -0.183 | | -0.083 | | -0.138 | | | -0.047 | | | -0.135 | | | -0.078 | | |  |  |
| Arachidic acid | 0.101 | | -0.186 | | 0.126 | | | -0.181 | | | 0.110 | | | -0.155 | | |  |  |
| Docosanoic acid | -0.149 | | -0.108 | | -0.125 | | | -0.104 | | | -0.126 | | | -0.097 | | |  |  |
| Eicosaenoic acid | -0.127 | | -0.009 | | -0.120 | | | -0.012 | | | -0.106 | | | -0.013 | | |  |  |
| Hexadecenoic acid | -0.049 | | -0.042 | | -0.057 | | | -0.049 | | | -0.056 | | | -0.044 | | |  |  |
| Hexadecanoic acid | 0.311 | | -0.138 | | 0.291 | | | -0.176 | | | 0.296 | | | -0.154 | | |  |  |
| Lignoceric acid | 0.262 | | 0.267 | | 0.218 | | | 0.289 | | | 0.227 | | | 0.316 | | |  |  |
| Octadecanoic acid | -0.003 | | 0.250 | | -0.010 | | | 0.209 | | | -0.020 | | | 0.186 | | |  |  |
| Octadecenoic acid | -0.061 | | -0.051 | | -0.073 | | | -0.040 | | | -0.092 | | | -0.033 | | |  |  |
| Tetracosenic acid | 0.070 | | -0.078 | | 0.057 | | | -0.106 | | | 0.043 | | | -0.114 | | |  |  |
| Tetradecanoic acid | 0.083 | | 0.115 | | 0.097 | | | 0.123 | | | 0.082 | | | 0.099 | | |  |  |

Note**:** Qgcomp was used to estimate the joint effect of multiple fatty acids on fetal growth trajectories; weights represent the estimated contribution of each fatty acid to the overall effect; adjustment models included covariates such as maternal age, body mass index, years of education, smoking/drinking, marriage, and han ethnic groups; the IPTW adjustment model used propensity-score-based inverse probability-of-treatment weighting to account for potential confounders; Positive weighting indicates a positive association with the outcome and negative weighting indicates a negative association with the outcome.

**eTable 8.** *P* values for departure from linearity in generalized additive models (GAM) in CLIMB study.

| Fatty acids |  | | | Male | | Female | | | | | | | | |  |  |  |  |
| --- | --- | --- | --- | --- | --- | --- | --- | --- | --- | --- | --- | --- | --- | --- | --- | --- | --- | --- |
|  | **stable falling vs. stable increasing**  **(n=118 vs. n=394) 1 vs.2** | | | **stable falling vs. stable increasing (n=46 vs. n=207) 1 vs.2** | | **stable falling vs. stable increasing**  **(n=72 vs. n=187)1 vs.2** | | | | | | | | |  |  |  |  |
| Docosahexaenoic acid | | 0.583 | 0.842 | | | | 0.448 | | | | | | |  |  |  |  |  |
| Eicosapentaenoic acid | 0.403 | | | 0.315 | | 0.400 | | | | | | | | |  |  |  |  |
| α-linolenic acid | 0.633 | | | 0.122 | | 0.382 | | | | | | | | |  |  |  |  |
| Docosapentenoic acid | 0.206 | | | 0.836 | | 0.398 | | | | | | | | |  |  |  |  |
| γ-linolenic acid | 0.475 | | | 0.691 | | 0.248 | | | | | | | | |  |  |  |  |
| Arachidonic acid | 0.787 | | | 0.199 | | 0.544 | | | | | | | | |  |  |  |  |
| Docosatetraenoic acid | 0.843 | | | 0.662 | | 0.638 | | | | | | | | |  |  |  |  |
| Eicosadienoic acid | 0.405 | | | 0.243 | | 0.574 | | | | | | | | |  |  |  |  |
| Eicosatrienoic acid | 0.989 | | | 0.662 | | 0.470 | | | | | | | | |  |  |  |  |
| Linoleic acid | 0.332 | | | 0.584 | | 0.921 | | | | | | | | |  |  |  |  |
| Arachidic acid | 0.993 | | | 0.684 | | 0.576 | | | | | | | | |  |  |  |  |
| Docosanoic acid | 0.485 | | | 0.923 | | 0.701 | | | | | | | | |  |  |  |  |
| Eicosaenoic acid | 0.052 | | | 0.161 | | **<0.001 **** | | | | | | | | | | | |  |
| Hexadecenoic acid | 0.814 | | | 0.364 | | 0.700 | | | | | | | | |  |  |  |  |
| Hexadecanoic acid | 0.721 | | | 0.979 | | 0.770 | | | | | | | | |  |  |  |  |
| Lignoceric acid | 0.222 | | | 0.133 | | 0.393 | | | | | | | | |  |  |  |  |
| Octadecanoic acid | 0.541 | | | 0.992 | | 0.126 | | | | | | | | |  |  |  |  |
| Octadecenoic acid | 0.273 | | | 0.999 | | **0.013*** | | | | | | | | | | |  |  |
| Tetracosenic acid | 0.460 | | | 0.408 | | 0.323 | | | | | | | | |  |  |  |  |
| Tetradecanoic acid | 0.610 | | | 0.865 | | 0.900 | | | | | | | | |  |  |  |  |
|  | **high stable increasing vs. stable increasing (n=92 vs. n=394)3 vs.2** | | | **high stable increasing vs. stable increasing (n=59 vs. n=207)3 vs.2** | | **high stable increasing vs. stable increasing (n=33 vs. n=187)3 vs.2** | | | | | | | | |  |  |  |  |
| Docosahexaenoic acid | | 0.983 | 0.519 | | | | 0.710 | | | | | | | | |  |  |  |
| Eicosapentaenoic acid | 0.812 | | | 0.651 | **0.004 **** | | | | | | | | | | | | | |
| α-linolenic acid | 0.854 | | | 0.625 | | 0.987 | | | | | | | | |  |  |  |  |
| Docosapentenoic acid | 0.852 | | | 0.290 | | 0.663 | | | | | | | | |  |  |  |  |
| γ-linolenic acid | 0.104 | | | 0.258 | | 0.056 | | | | | | | | |  |  |  |  |
| Arachidonic acid | 0.257 | | | 0.293 | | 0.186 | | | | | | | | |  |  |  |  |
| Docosatetraenoic acid | 0.294 | | | 0.277 | | 0.691 | | | | | | | | |  |  |  |  |
| Eicosadienoic acid | 0.279 | | | 0.642 | | 0.752 | | | | | | | | |  |  |  |  |
| Eicosatrienoic acid | 0.763 | | | 0.322 | | 0.965 | | | | | | | | |  |  |  |  |
| Linoleic acid | 0.215 | | | 0.344 | | 0.285 | | | | | | | | |  |  |  |  |
| Arachidic acid | 0.634 | | | 0.173 | | 0.903 | | | | | | | | |  |  |  |  |
| Docosanoic acid | 0.837 | | | 0.834 | | 0.525 | | | | | | | | |  |  |  |  |
| Eicosaenoic acid | 0.396 | | | 0.455 | | 0.976 | | | | | | | | |  |  |  |  |
| Hexadecenoic acid | 0.782 | | | 0.931 | | 0.974 | | | | | | | | |  |  |  |  |
| Hexadecanoic acid | 0.389 | | | 0.237 | | 0.709 | | | | | | | | |  |  |  |  |
| Lignoceric acid | 0.666 | | | 0.203 | | 0.628 | | | | | | | | |  |  |  |  |
| Octadecanoic acid | 0.165 | | | **0.044 *** | | | | | 0.507 | | |  |  |  |  |  |  |  |
| Octadecenoic acid | 0.427 | | | 0.392 | | 0.930 | | | | | | | | |  |  |  |  |
| Tetracosenic acid | 0.849 | | | 0.678 | | 0.868 | | | | | | | | |  |  |  |  |
| Tetradecanoic acid | 0.315 | | | 0.336 | | 0.073 | | | | | | | | |  |  |  |  |
|  | **dramatically falling vs. stable increasing (n=53 vs. n=394)4 vs.2** | | | **dramatically falling vs. stable increasing (n=29 vs. n=207) 4 vs.2** | | **dramatically falling vs. stable increasing**  **(n=24 vs. n=187) 4 vs.2** | | | | | | | | |  |  |  |  |
| Docosahexaenoic acid | | 0.070 | 0.902 | | | | 0.840 | | | | | | |  |  |  |  |  |
| Eicosapentaenoic acid | 0.123 | | | 0.229 | | 0.877 | | | | | | | | |  |  |  |  |
| α-linolenic acid | **0.046*** | | | 0.171 | | 0.458 | | | | | | | | |  |  |  |  |
| Docosapentenoic acid | 0.498 | | | 0.065 | | 0.606 | | | | | | | | |  |  |  |  |
| γ-linolenic acid | 0.154 | | | 0.153 | | 0.116 | | | | | | | | |  |  |  |  |
| Arachidonic acid | 0.769 | | | 0.495 | | 0.932 | | | | | | | | |  |  |  |  |
| Docosatetraenoic acid | 0.509 | | | 0.202 | | 0.727 | | | | | | | | |  |  |  |  |
| Eicosadienoic acid | 0.762 | | | **0.011*** | | | | | 0.644 | | |  |  |  |  |  |  |  |
| Eicosatrienoic acid | 0.977 | | | 0.313 | | 0.595 | | | | | | | | |  |  |  |  |
| Linoleic acid | 0.937 | | | 0.829 | | 0.867 | | | | | | | | |  |  |  |  |
| Arachidic acid | 0.335 | | | **0.004**** | | | | | | 0.658 |  |  |  |  |  |  |  |  |
| Docosanoic acid | 0.849 | | | **0.042*** | | | | 0.994 | | | | |  |  |  |  |  |  |
| Eicosaenoic acid | 0.335 | | | 0.062 | | 0.223 | | | | | | | | |  |  |  |  |
| Hexadecenoic acid | 0.634 | | | 0.228 | | 0.583 | | | | | | | | |  |  |  |  |
| Hexadecanoic acid | 0.519 | | | 0.179 | | 0.483 | | | | | | | | |  |  |  |  |
| Lignoceric acid | 0.436 | | | 0.060 | | 0.829 | | | | | | | | |  |  |  |  |
| Octadecanoic acid | 0.755 | | | 0.648 | | 0.463 | | | | | | | | |  |  |  |  |
| Octadecenoic acid | 0.247 | | | 0.403 | | 0.181 | | | | | | | | |  |  |  |  |
| Tetracosenic acid | 0.535 | | | 0.934 | | 0.156 | | | | | | | | |  |  |  |  |
| Tetradecanoic acid | 0.412 | | | **0.049*** | | | | **0.028 *** | | | | | | | | |  |  |

Note**:** The value is expressed as p-value; adjusted models included covariates maternal age, body mass index, years of education, smoking/drinking, marriage, and han ethnic groups; analyses were stratified according to fetal gender (male vs. female) to explore potential sex-specific differences; **p* < 0.05, ***p* < 0.01, indicates statistical significance.





**eFigure 1**: The directed acyclic graph (DAG) for all potential confounders considered in the CLIMB study.

**eFigure 2:** Relationship between prenatal fatty acids level (mg/L) and trajectory groups in the generalized additive models (GAM), both the fitted and 95% confidence interval lines are presented in CLIMB study (stable falling vs. stable increasing).

**eFigure 3:** Relationship between prenatal fatty acids level (mg/L) and trajectory groups in the generalized additive models (GAM), both the fitted and 95% confidence interval lines are presented in CLIMB study (high stable increasing vs. stable increasing).

**eFigure 4:** Relationship between prenatal fatty acids level (mg/L) and trajectory groups in the generalized additive models (GAM), both the fitted and 95% confidence interval lines are presented in CLIMB study (dramatically falling vs. stable increasing).
